# Microbial Hydroxysteroid Dehydrogenases: From Alpha to Omega

**DOI:** 10.3390/microorganisms9030469

**Published:** 2021-02-24

**Authors:** Heidi L. Doden, Jason M. Ridlon

**Affiliations:** 1 Microbiome Metabolic Engineering Theme, Carl R. Woese Institute for Genomic Biology, Urbana, IL 61801, USA; hdoden2@illinois.edu; 2 Department of Animal Sciences, University of Illinois at Urbana-Champaign, Urbana, IL 61801, USA; 3 Division of Nutritional Sciences, University of Illinois at Urbana-Champaign, Urbana, IL 61801, USA; 4 Cancer Center of Illinois, University of Illinois at Urbana-Champaign, Urbana, IL 61801, USA; 5 Department of Microbiology and Immunology, Virginia Commonwealth University School of Medicine, Richmond, VA 23298, USA

**Keywords:** hydroxysteroid dehydrogenase, sterolbiome, cholesterol, bile acid, cortisol, androgen, deoxycholic acid

## Abstract

Bile acids (BAs) and glucocorticoids are steroid hormones derived from cholesterol that are important signaling molecules in humans and other vertebrates. Hydroxysteroid dehydrogenases (HSDHs) are encoded both by the host and by their resident gut microbiota, and they reversibly convert steroid hydroxyl groups to keto groups. Pairs of HSDHs can reversibly epimerize steroids from α-hydroxy conformations to β-hydroxy, or β-hydroxy to ω-hydroxy in the case of ω-muricholic acid. These reactions often result in products with drastically different physicochemical properties than their precursors, which can result in steroids being activators or inhibitors of host receptors, can affect solubility in fecal water, and can modulate toxicity. Microbial HSDHs modulate sterols associated with diseases such as colorectal cancer, liver cancer, prostate cancer, and polycystic ovary syndrome. Although the role of microbial HSDHs is not yet fully elucidated, they may have therapeutic potential as steroid pool modulators or druggable targets in the future. In this review, we explore metabolism of BAs and glucocorticoids with a focus on biotransformation by microbial HSDHs.

## 1. Introduction

**Figure 1:**
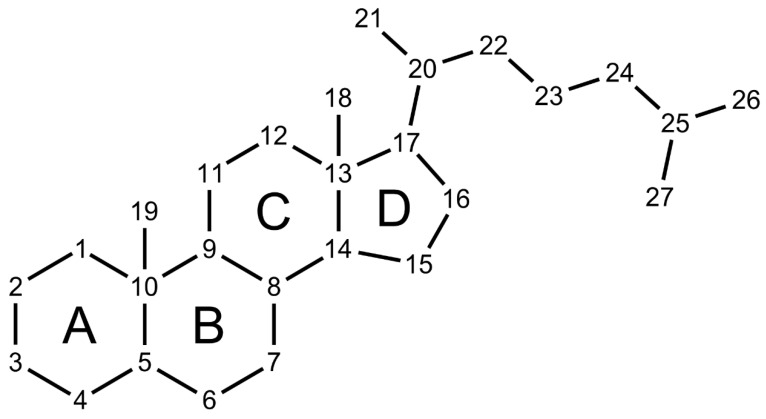
Steroid structure. Steroids have a cyclopentanoperhydrophenanthrene ring structure. Cholesterol, the precursor to human steroid hormones, contains 27 carbons, while the major classes of steroid hormones contain the following: C_24_ bile acids, C_19_ androgens, C_18_ estrogens, and C_21_ glucocorticoids, mineralocorticoids, and progestogens.

Steroid hormones are signaling molecules derived from cholesterol that include glucocorticoids, mineralocorticoids, androgens, estrogens, progestogens, and bile acids (BAs) [1]. Steroid hormones are essential for the regulation of various physiological processes, such as metabolism, salt and water balance, reproduction, inflammation, and stress response [2]. These cholesterol-derived molecules are synthesized in the human adrenal glands, gonads, placenta, and liver [3,4]. All steroids have a cyclopentanoperhydrophenanthrene ring structure, composed of three six-carbon rings denoted A, B, and C along with a five-carbon D ring (Figure 1), with differing hydroxyl groups and side-chains [1]. Hydroxysteroid dehydrogenases (HSDH) are an important class of enzyme expressed by both host tissues and host-associated microbiota that modify the hydroxyl groups on steroids. These small modifications to steroids greatly impact their physicochemical properties and can change the steroid solubility, toxicity, host receptor affinity, and ability to activate or inhibit host receptors [5,6,7,8]. The current review focuses on the importance of gut microbial HSDHs in cholesterol, BA, and glucocorticoid metabolism. 

## 2. Hydroxysteroid Dehydrogenases

### 2.1. Hydroxysteroid Dehydrogenase Function

Hydroxysteroid dehydrogenases are nicotinamide adenine dinucleotide (phosphate) (NAD(P)(H))-dependent oxidoreductases that catalyze the reversible conversion of hydroxyl groups to keto groups on steroids [9]. HSDHs are regio- and stereospecific, meaning they are specific for the hydroxyl position on the steroid (C-3 vs. C-7) and for the orientation (α vs. β) of the hydroxyl group, respectively [5]. Pairs of HSDHs can convert steroids from the α-orientation, through an oxo-intermediate, to the epimerized β-orientation and vice versa.

Hydroxysteroid dehydrogenases are found in both host and microbial genomes, although more is known about the physiological function of host hydroxysteroid dehydrogenases, which are typically abbreviated HSDs in literature. In this review, host hydroxysteroid dehydrogenases are denoted “HSD” while bacterial enzymes are denoted “HSDH”. Host HSDs are key enzymes in the biosynthesis of steroids in steroidogenic tissues [10]. They also function to activate or inactivate steroids in peripheral tissues, thus regulating local concentrations of steroid hormones [5]. Even though host HSDs catalyze reversible reactions in vitro, they typically function primarily in one direction in vivo on the basis of cofactor balance: either as dehydrogenases or as reductases [11].

Host HSDs are druggable targets important in the treatment of endocrine-dependent disorders, including cancers [12]. Host-associated microbial HSDHs may also serve as pharmacological targets or, alternatively, may be enriched in the host through engineering and delivering probiotic bacteria with rational sterolbiome phenotypes. One recent example involves identification of a cholesterol 3β-HSDH involved in conversion of cholesterol to coprostanol, the enrichment of which may be important as a probiotic approach to reducing serum cholesterol [13].

### 2.2. Structural Biology of Hydroxysteroid Dehydrogenases

Hydroxysteroid dehydrogenases belong to one of the following three large and diverse protein superfamilies: short-chain dehydrogenase/reductase (SDR), medium-chain dehydrogenase/reductase (MDR), or aldo-keto reductase (AKR) [5,14]. Many SDR and MDR family hydroxysteroid dehydrogenases have been identified in the gut microbiome [14,15,16,17]. HSDHs in the AKR superfamily are generally found within mammals [12], although microbial AKR family HSDHs have been reported [18].

The SDR superfamily is one of the largest, containing proteins spanning all three domains of life [19]. SDR proteins have highly diverse substrate specificities, ranging from sugars to dyes to steroids [20]. Members of this superfamily are non-metalloenzymes and typically 250 amino acids in length [5]. Due to the dependence of dehydrogenase/reductase enzymes on NAD(P)(H) to carry out redox reactions, SDR proteins contain a Rossmann fold domain for binding cofactors. This domain consists of 6–7 β-strands with 3–4 peripheral α-helices on either side [21,22]. Typically, the Rossmann fold domain is located near the N-terminus of SDR proteins, while the C-terminus binds substrates [20]. Most SDR members have a conserved Tyr, Ser, and Lys at the catalytic site. The overall folding pattern is closely conserved across the superfamily, while amino-acid sequence varies greatly [22]. This causes great difficulty in predicting substrate specificities by amino-acid homology search alone. HSDHs within the SDR superfamily include but are not limited to host 11β-HSD and 17β-HSD [5], and various microbial BA 12α-HSDHs [23], 12β-HSDH [24], 3α/β-HSDHs [17], and glucocorticoid 20β-HSDH [15].

The MDR family is similar to the SDR family both in number of members and in function, although their structures have marked differences. MDR proteins contain Rossmann fold domains for NAD(P)(H) binding like SDRs, but they are ~350 residues long and many are metal-dependent [25]. They are typically dimeric or tetrameric and many contain a catalytic zinc ion, sometimes along with a structural zinc ion, while others are non-zinc-containing [26]. The zinc-containing MDRs share a strictly conserved Gly, His, and Glu for zinc binding [27]. MDR family HSDHs include host BA 3β-HSD [26] and microbial glucocorticoid 20α-HSDH [14,16].

AKRs are NAD(P)(H)-dependent oxidoreductases acting on carbonyl groups or double bonds and are ~320 amino acids long. They are monomeric with diverse substrate recognition, including steroids, monosaccharides, and isoflavonoids. An ordered bi–bi kinetic mechanism has been shown for multiple AKR family members, where the cofactor is first to bind and last to leave [28]. Most have a conserved active site with residues Asp, Lys, Tyr, and His. Examples of members of this superfamily involved in steroid metabolism are human 3α-HSD [29], human 20α-HSD [30], and bacterial BA 3β-HSDH [18].

## 3. Bile Acid Metabolism

### 3.1. Host Bile Acid Synthesis and Signaling

Bile acids are amphipathic C_24_ steroids that play an important role in host nutrition [31]. They are essential for solubilization and later absorption of cholesterol, dietary fatty acids, triglycerides, and lipid-soluble vitamins A, D, E, and K. Bile acids assemble into mixed micelles, forming a hydrocarbon interior in order to solubilize these molecules [31,32].

**Figure 2:**
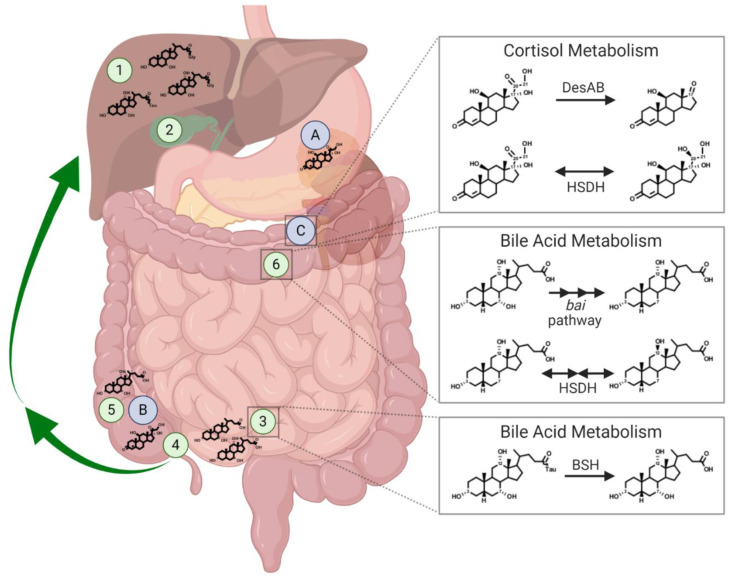
Synthesis and microbial metabolism of bile acids and cortisol. (1) The bile acids (BAs) cholic acid (CA) and chenodeoxycholic acid (CDCA) are synthesized and conjugated to glycine (Gly) or taurine (Tau) in the liver. (2) They are then stored in the gallbladder until they are released in response to a meal. (3) Microbial deconjugation of amino acids, catalyzed by bile salt hydrolase (BSH), primarily occurs in the small intestine. (4) BAs are taken up in the terminal ileum and undergo enterohepatic circulation back to the liver indicated by green arrows. (5) About 5% of BAs are not recycled and proceed to the colon. (6) Gut microbiota residing in the colon can 7α-dehydroxylate CA or CDCA to secondary BAs in a pathway encoded by the BA-inducible (*bai*) operon. Microbial hydroxysteroid dehydrogenases (HSDHs) interconvert BA hydroxyl groups between the α- and β-conformations through an oxo-intermediate. (A) Cortisol is synthesized in the adrenal glands. (B) Cortisol and its derivatives are principally excreted in urine; however, low levels are secreted in bile and enter the gut. (C) In the gut, cortisol can be side-chain cleaved by microbiota encoding steroid-17,20-desmolase (DesAB) or reduced to 20α- or 20β-dihydrocortisol by HSDHs.

Bile acid biosynthesis occurs in the liver and begins with the rate-limiting step of cholesterol 7α-hydroxylation by cytochrome P450 7α-hydroxylase (CYP7A1) in hepatocytes (Figure 2) [31,33]. While other carbon positions on cholesterol can be hydroxylated first (C-24, C-25, C-26, C-27), the classical pathway initiates through C-7 hydroxylation catalyzed by CYP7A1 [34,35]. The next step alters the ring structure through conversion to 3-oxo-Δ^4^ by 3β-hydroxy-Δ^5^-C_27_-steroid oxidoreductase (HSD3B7) [34,36,37]. After HSD3B7 action, the intermediate is converted by 12α-hydroxylase (CYP8B1) if the final product contains a 12α-hydroxyl group. Ensuing steps involve additional modification to the ring structure by AKR1D1 and AKR1C1 [37]. Then, mitochondrial sterol 27-hydroxylase (CYP27A1) oxidizes the side-chain, followed by removal of three carbon atoms beginning with activation of the sterol by BA coenzyme A (CoA) synthase [34,38,39]. Subsequent reactions are catalyzed by 2-methylacyl-CoA racemase, branched-chain acyl-CoA oxidase, d-bifunctional protein, and peroxisomal thiolase 2, which cleaves the C-24–C-25 bond [34,37]. The final step in BA biosynthesis is conjugation of the BA-CoA intermediate to either glycine or taurine, catalyzed by BA CoA:amino acid *N*-acyltransferase [34,40].

Conjugated BAs, called “bile salts” due to their ionized state at physiological pH, have increased solubility and greater amphipathicity. The biosynthetic pathway results in the formation of conjugated cholic acid (CA; 3α,7α,12α-hydroxy) or chenodeoxycholic acid (CDCA; 3α,7α-hydroxy) with their relative proportions determined by levels of 12α-hydroxylase in the liver [33,34]. The ratio of taurine- to glycine-conjugated BAs is dependent on diet in humans. A high-protein diet results in greater taurine conjugation, while vegetarian diets lead to more glycine conjugation [33]. CA and CDCA are the primary BAs produced in humans, whereas other vertebrates produce bile salts that differ in ring hydroxylation pattern, as well as side-chain length and functional groups. The main classes are C_24_ BAs, C_27_ BAs, and C_27_ bile alcohols [41]. C_24_ BAs are common in all vertebrates, but with differing hydroxylation patterns. For example, mice produce CA and convert CDCA to muricholic acids (3,6,7-hydroxy) via hydroxylation and epimerization at C-6. C_27_ bile alcohols are typically synthesized in fish [42] and amphibians, while C_27_ BAs are present in reptiles and birds [41].

Once synthesized, conjugated BAs are actively transported out of hepatocytes into the bile duct. Conjugated BAs are stored in the gallbladder until the gallbladder is emptied into the duodenum in response to a meal [43]. Conjugated bile salts form mixed micelles with cholesterol, lipid-soluble vitamins, and dietary lipids throughout the small intestine. In the ileum, a sodium-dependent transporter (IBAT) takes up BAs into ileocytes [44]. From ileocytes, they are exported by organic solute transporter OSTα/β [45,46] into the portal vein, where they circulate back to the liver in a process known as enterohepatic circulation [47]. However, ~500 mg of BAs each day are not taken up in the ileum and progress to the colon where they encounter gut microbiota [37]. Microbial metabolites of BAs can be passively absorbed in the colon, travel through the portal vein, and join the recycled host-derived BAs in the liver. Thus, the biliary pool consists of both host- and microbiota-derived BAs that are re-conjugated and, in some species, 7-hydroxylated, as they return to the liver [48]. 

In addition to the digestive function of BAs, they are now known to act as hormone signaling molecules. BAs are involved in regulation of their own biosynthesis, as well as energy, glucose, and lipid metabolism [43]. Farnesoid X receptor (FXR, NR1H4) is a BA-activated nuclear receptor expressed in tissues such as liver, intestine, and kidney [49,50]. FXR regulates BA biosynthesis and enterohepatic circulation through many mechanisms. The FXR/SHP (small heterodimer partner) pathway of regulation involves the inhibition of *CYP7A1*, the rate-limiting step in BA formation. FXR induces the nuclear receptor, SHP, which inhibits liver-related homolog-1 (LRH-1) and hepatocyte nuclear factor 4α (HNF4α), both leading to inhibition of *CYP7A1* transcription [51,52,53]. Another pathway involves FXR, fibroblast growth factor 19 (FGF19), and FGF receptor 4 (FGFR4), which also results in inhibition of *CYP7A1.* Before recirculation back to the liver, BAs stimulate intestinal FXR, which induces FGF19 synthesis in ileocytes [54]. FGF19 is transported to the liver, where it binds FGFR4 and activates the *c-jun* N-terminal kinase (JNK) 1/2 signaling cascade, leading to downregulation of *CYP7A1* [33,55].

Pregnane X receptor (PXR) and vitamin D receptor (VDR) are both nuclear receptors activated by microbial-derived BAs that also lead to the binding of *CYP7A1* promoter and repression of CYP7A1 [8,56,57,58]. Takeda G-protein receptor 5 (TGR5) is a G-protein-coupled receptor for BAs that is expressed in intestinal and biliary epithelial cells among other cell types [59,60]. TGR5 has widespread effects throughout the body, including regulation of intestinal motility [61]. Taurine-conjugated BAs activate TGR5 more effectively than unconjugated or glycine-conjugated BAs [62]. TGR5 signaling can activate epidermal growth factor receptor (EGFR) [63]. EGFR is also a BA receptor that, once bound, initiates a signaling pathway ending in inhibition of CYP7A1 [43,64]. In the gut, primary bile salts can be microbially biotransformed to dozens of metabolites whose concentrations and affinities can impact host physiological response in the intestine.

### 3.2. Microbial Bile Acid Metabolism

Bile acids that enter the colon are metabolized by gut microbiota through a combination of de(re)conjugation, 7α/β-dehydroxylation, and epimerization (Figure 2). The first step of microbial BA metabolism, known as deconjugation, mainly occurs in the small intestine and involves the hydrolysis of the C-24 *N*-acyl bond linking the conjugated amino acid to the BA. This reaction is catalyzed by bile salt hydrolase (BSH) encoded by diverse microbiota, including *Clostridium* [65,66], *Bacteroides* [67,68], Lactobacillaceae [69], *Bifidobacterium* [70,71], *Enterococcus* [72], and archaea [73]. BSHs have differing substrate specificity and subunit size, but often have conserved active site Cys, Arg, Asp, Asn, and another Arg [74]. BSHs have a pH optimum of 5–6 and are typically intracellular [65,70], although activity has been reported extracellularly in some cases [66]. Interestingly, re-conjugation of BAs by gut microbiota has recently been observed with unique amino acids: Phe, Tyr, and Leu [75].

There are multiple hypotheses on the evolutionary role of BSH in microbial fitness: interspecies competition, detoxification, and release of an energy source. Deconjugated BAs are more toxic than conjugated bile salts to some bacterial species; thus, deconjugation may serve a competitive function to inhibit other bacteria [4]. However, the reverse may also be true. Some bacteria are more sensitive to conjugated BAs and, thus, BSH may help them detoxify their environment [76]. Amino acids released from deconjugation could be an important energy source for certain microbiota, such as *Clostridium* that can utilize amino acids through Stickland fermentation [77].

Deconjugated primary BAs can be 7-dehydroxylated by a select few species within the gut, including *Clostridium scindens*, *C. hylemonae*, and *C. hiranonis* (now reclassified as *Peptacetobacter hiranonis*) [4,78,79,80]. Through this process, the primary BAs CA and CDCA are converted to “secondary” deoxycholic acid (DCA; 3α,12α-hydroxy) and lithocholic acid (LCA; 3α-hydroxy), respectively. Although so few species encode the 7α-dehydroxylation pathway, secondary BAs make up the majority of excreted BAs [74,81,82], meaning these microbiota have extensive dehydroxylation capacity.

The 7-dehydroxylation pathway is encoded by the polycistronic BA-inducible (*baiABCDEFGHI*) operon [4,83,84]. The first step is the import of unconjugated primary BAs by a BA transporter BaiG [85]. Next, ligation of CoA to the unconjugated BA is catalyzed by BA CoA ligase encoded by *baiB*, requiring ATP and Mg^2+^ [86]. Then, the 3α-hydroxyl group is oxidized by BaiA [87]. Three *baiA* genes from *C. scindens* have been reported in *C. scindens* VPI 12708, although completion of the *C. scindens* American Type Culture Collection (ATCC) 35704 genome revealed the presence of only two, with *baiA2* located in the *bai* operon [88,89,90,91]. These enzymes are NAD(H)-dependent BA 3α-HSDHs that are specific for BA-CoA conjugates [87]. BaiCD is an NADH:flavin-dependent oxidoreductase that creates a C-4=C-5 double bond on 7α-hydroxy BA intermediates, while BaiH has the same function on 7β-hydroxy BAs [92]. CoA is then hydrolyzed by BaiF or BaiK and transferred without requirement of ATP to an incoming primary BA [93]. Subsequent 7α-dehydration is the rate-limiting step in the pathway, catalyzed by the *baiE* product [94]. 7β-Dehydration is predicted to be carried out by BaiI [95]. Recently, a recombinant flavoprotein encoded by *baiN*, which is not a part of the *bai* operon, was shown to convert 3-dehydro-DCA to a product 4 amu less than the substrate [96]. Further characterization is necessary, but this suggests that *baiN* may catalyze reduction of both Δ^4^ and Δ^6^-intermediates following 7-dehydration [96]. Alternatively, BaiCD and BaiH were reported to be sufficient for C-4=C-5 and C-6=C-7 metabolism in the oxidative and reductive arms of the pathway [97]. The final step in the pathway, converting the 3-oxo intermediate to a secondary BA, is likely to be carried out by the products of one or both copies of *baiA* [98]. The BA exporter is not yet known [4]. However, two genes co-localized with *baiN* have been proposed, but not yet confirmed, to catalyze the final reaction and BA export, named BaiO and BaiP, respectively [99]. Several additional candidate export proteins were identified through transcriptomic analysis of *C. scindens* ATCC 35704 after BA induction [91]. 

The 7α/β-dehydroxylation pathway results in a net two-electron reduction, meaning a net of one NAD^+^ is produced when a primary BA is used as an electron acceptor [74]. The 7α/β-dehydroxylation pathway is likely coupled to glucose metabolism, benefitting 7α/β-dehydroxylating bacteria [91]. The pathway may serve another function in producing secondary BAs, which are more hydrophobic and toxic to gut bacteria, to regulate the growth of competing gut microbiota [7,100]. For example, DCA has a minimum inhibitory concentration tenfold lower than CA against many *Lactobacillus* and *Bifidobacterium* species [100].

**Figure 3:**
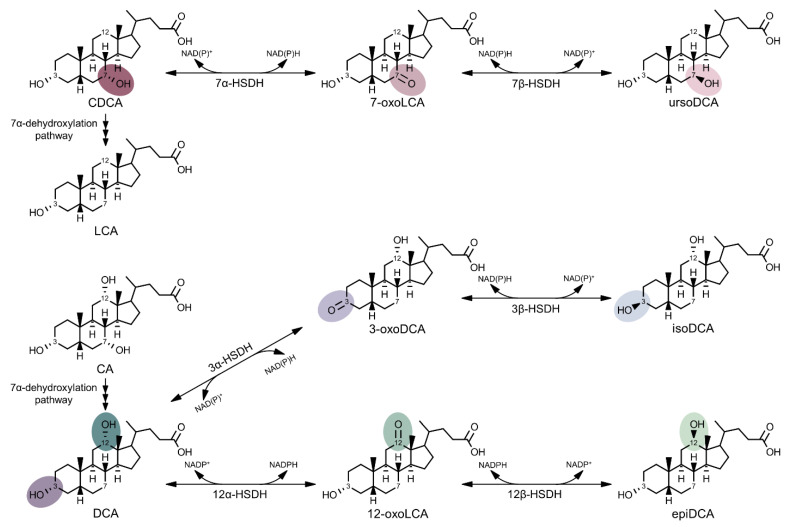
Microbial bile acid hydroxysteroid dehydrogenase metabolism. After deconjugation by bile salt hydrolase, the primary bile acids (BAs) chenodeoxycholic acid (CDCA) and cholic acid (CA) can be 7α-dehydroxylated or reversibly biotransformed by NAD(P)(H)-dependent hydroxysteroid dehydrogenases (HSDHs). CDCA is converted to the oxo-intermediate, 7-oxolithocholic acid (7-oxoLCA), and further to ursoDCA (UDCA) in the urso-BA pathway catalyzed by 7α- and 7β-HSDH. The secondary BAs lithocholic acid (LCA) and deoxycholic acid (DCA) are produced through the multi-step 7α-dehydroxylation of CDCA and CA, respectively. 3α-HSDH biotransforms DCA into 3-oxoDCA, and 3β-HSDH converts 3-oxoDCA to isoDCA in the iso-BA pathway. DCA is converted to 12-oxoLCA by 12α-HSDH and from 12-oxoLCA to epiDCA by 12β-HSDH. HSDHs can recognize other BAs with the correct hydroxyl group position and orientation beyond those depicted.

Both primary and secondary BAs can be oxidized and epimerized at position C-3, C-7, and/or C-12 reversibly from the α-orientation to an oxo-intermediate and further to the β-orientation by microbial HSDHs. Epimerized BAs have specific nomenclature: those containing 3β-hydroxyl groups are iso-BAs, while 7β- and 12β-BAs are recommended to be denoted epi-BAs preceded by the hydroxyl position, according to Hofmann et al. (1992) [101]. However, 7β-BAs are generally accepted to be named urso-BAs. For simplicity in this review, each prefix refers to only one of the β-hydroxyl positions: iso for 3β-, urso for 7β-, and epi for 12β-hydroxyl (Figure 3). Similarly to humans, mouse α-muricholic acid (3α,6β,7α-hydroxy) and β-muricholic acid (3α,6β,7β-hydroxy) can be oxidized and epimerized to ω-muricholic acid (3α,6α,7β-hydroxy) via a 6-oxo-intermediate [102]. Numerous microbiota are capable of oxidoreduction of BAs, including *Eggerthella lenta* [103], *C. scindens* [23,87], *C. hiranonis* [23], *C. hylemonae* [23], *Escherichia coli* [104], and *Bacteroides fragilis* [105].

### 3.3. Microbial Bile Acid Hydroxysteroid Dehydrogenases

Microbial HSDHs catalyze the NAD(P)(H)-dependent oxidation and reduction of hydroxyl groups on BAs in the gut (Figure 3). Human interest in ursodeoxycholic acid (UDCA; 3α,7β-hydroxy) has a long and fascinating history. Asiatic black bear bile has been used in traditional Chinese medicine to treat disease for over 1000 years [106]. In the early 1900s, a BA was isolated from polar bear bile and, later, the same BA was crystallized from the American black bear. This BA was named ursodeoxycholic acid after the Latin name *ursus* [107]. UDCA makes up about 3–4% of the human BA pool but, in contrast to bear bile, is a secondary BA in humans [108,109]. UDCA and other urso-BAs are produced by combined microbial 7α-HSDH and 7β-HSDH activity in the human gut. Both microbial 7α- and 7β-HSDHs are typically NADP(H)-dependent, and they frequently exhibit specificity for dihydroxy-BAs (e.g., CDCA and UDCA) over trihydroxy-BAs (e.g., CA and UCA) [104,105,110,111,112,113,114], although exceptions have been reported [115,116].

Urso-BAs are more hydrophilic and less toxic both to microbiota and to the host than DCA or LCA [7]. Indeed, DCA and LCA are involved in various diseases, such as cancers of the colon and liver [117,118,119,120]. UDCA is currently approved for treatment of biliary disorders [121], is being studied for both chemoprevention and chemotherapy of various cancers [108,122], and is undergoing clinical trials as part of a combination chemotherapy for colorectal cancer (clinicaltrials.gov identifier: NCT00873275). Its mechanism of action likely involves the displacement of more toxic BAs in the BA pool and its choleretic effect of inducing secretion of BAs from the liver [123]. However, UDCA can be 7β-dehydroxylated by certain gut microbiota or isomerized back to 7α-hydroxy prior to 7α-dehydroxylation [124,125]. 7β-Dehydroxylation of UDCA forms LCA, which may explain various toxicities associated with UDCA treatment [126].

The iso-BA pathway is catalyzed by the paired action of BA 3α- and BA 3β-HSDH. Generally, 3α-HSDHs utilize NAD(H), whereas 3β-HSDHs require NADP(H). They also usually prefer dihydroxy-BAs (derivatives of DCA or CDCA) over trihydroxy-BAs (derivatives of CA) [17,18,112,127]. BA 7α-dehydroxylating bacteria express a 3α-HSDH (BaiA) that differs greatly in substrate specificity as it reacts with CoA conjugates, not free BAs [87]. Iso-BAs are present ranging from 0% to about 20% of the total BA pool in the gut [109]. Iso-BAs have greatly decreased detergent nature and are thus less cytotoxic to gut microbiota, as well as the host, than DCA or LCA [6,17]. 3α/β-HSDHs may be of pharmaceutical use with respect to modulating the BA pool in favor of less toxic iso-BAs. Iso-BAs are intrinsically poor detergents and impede nutrient absorption. The liver epimerizes iso-BAs back to the 3α-hydroxyl form via a cytosolic 3β-HSDH [128]. Further studies are needed to determine the viability of developing strategies to favor iso-BAs. 

Compared to the iso- and urso-BA pathways, the least is known about the epi-BA pathway. While multiple 12α-HSDHs have been characterized [18,23,103,116,129,130], BA 12β-HSDH was only studied in cell extracts until the discovery of the first gene encoding this activity by our lab [24,131,132]. 12-Oxolithocholic acid (12-oxoLCA; 3α-hydroxy,12-oxo), the product of 12α-HSDH oxidation of DCA, is often one of the most abundant oxo-BAs found in human feces, at concentrations of about one half DCA in some studies [81,133,134]. Of note, levels of 12-oxoLCA were increased in rats with high incidence of tumors after being fed a diet high in corn oil or safflower oil [135]. Measurement of epi-BAs is rare in the literature. EpiDCA (3α,12β-hydroxy) was first identified in human feces by Eneroth et al. (1966) [136]. Recently, Franco et al. (2019) measured 3-oxo-12β-hydroxy-CDCA in humans, but little is known about concentrations of epiDCA or epiCA (3α,7α,12β-hydroxy) in feces [81]. EpiDCA has also been identified in the biliary bile of angelfish; hence, 12β-HSDH activity is likely present within the microbiome of diverse vertebrates [41].

Many gut microbial 12α-HSDHs have NADP(H) specificity [18,23,129,130], while others are NAD(H)-specific [116]. 12α-HSDHs generally have higher activity with free and dihydroxy-BAs than conjugated or trihydroxy-BAs [18,23,129]. The only gut microbial BA 12β-HSDH characterized to date, from *Clostridium paraputrificum* ATCC 25780, has affinity for NADP(H) and greater activity with dihydroxy-BAs [24,132]. Two additional 12β-HSDHs have been shown to react with 12-oxoLCA and epiDCA with NADP(H) as co-substrate, although their substrate specificities have not been fully characterized [24]. Interestingly, 12β-HSDH activity recognizing side-chain cleaved steroids derived from BAs has been observed in multiple environmental microorganisms. This activity is displayed by *Comamonas testosteroni* TA441 [137] and *Pseudomonas* sp. strain Chol1 [138] as they convert a 12-oxo-intermediate into 7α,12β-dihydroxy-androsta-1,4-diene-3,17-dione (12β-DHADD) in a cholic acid degradation pathway.

Epi-BAs are understudied compared to urso- and iso-BAs. Thus, their toxicity relative to secondary BAs is untested, although epiDCA and 12-oxoLCA are less hydrophobic than DCA according to LC–MS [24]. It is possible that isomerization of primary BAs to iso- or epi-BAs may impede formation of secondary BAs if they cannot be recognized by 7-dehydroxylation pathway enzymes. This could be of therapeutic importance because secondary BAs DCA and LCA are not only toxic to gut microbiota, but also to the human host.

Our knowledge of microbial HSDHs is largely limited to studies in humans and rodents. Notable recent studies extend to black bears in the search for HSDHs capable of forming UDCA [139]. There is a rich diversity of bile salts produced in vertebrates, such as pythocholic acid (16α-hydroxycholic acid; 3α,12α,16α-trihydroxy-5β-cholan-24-oic acid) found in snakes, which is a 16α-hydroxylated derivative of DCA [106,140]. Avicholic acid (3α,7α,16α-trihydroxy-5β-cholan-24-oic acid), found in birds, was identified in a drug screen as a TGR5 agonist [141]. An NAD(P)-dependent 16α-HSD was purified and characterized from rat kidney [142]; however, to our knowledge microbial 16α-HSDH activity has not yet been reported in snake or bird gastrointestinal content. 

### 3.4. Physiological Roles of Microbial Bile Acid Hydroxysteroid Dehydrogenases 

The physiological function of many microbial BA HSDHs remains unclear, although species and strain context seem likely to be important. In all cases, these redox reactions affect NAD(P)/NAD(P)H ratios, and BA oxo-groups provide substrates for disposal of excess reducing equivalents or acquisition of hydrides in order to detoxify molecular oxygen close to the gut mucosa. Oxidation and epimerization of BA α-hydroxyl groups to β-hydroxyl groups is also thought to function in detoxification by converting hydrophobic BAs to hydrophilic BAs that are less damaging to biological membranes [7,17]. For example, isoDCA has a minimum inhibitory concentration of more than double that of DCA against various Gram-negative *Bacteroides* and Gram-positive species [17]. In contrast, some HSDHs seem to favorably produce DCA from oxo-derivatives, suggesting they may function to maintain high concentrations of DCA in the environment [23].

Culture-based studies indicate that the oxidation and epimerization of primary BAs affects the extent of BA 7α-dehydroxylation [143]. There are several hypotheses that could explain this observation. First, there is currently a paucity of knowledge relating to substrate specificity of the BA transporter, BaiG, and whether oxo- and iso-BAs are efficiently imported. Our recent study indicates that 3,7-dioxoLCA is converted to CDCA and low levels of LCA by *C. scindens* [143], albeit to lower levels than CDCA addition, suggesting import is occurring. Second, BA 7α-dehydroxylating bacteria appear to lack significant 3β-HSDH activity and, as a result, iso-primary BAs (3β-hydroxy) are not substrates for the BA 7α-dehydroxylation pathway [143]. As noted above, the first oxidation step and the last reductive step in the BA 7α-dehydroxylation pathway are catalyzed by 3α-HSDH (BaiA). A 3β-hydroxyl group, thus, prevents key oxidation steps that lead to 7α-dehydration. Indeed, LCA was not observed in cultures of *C. scindens* VPI 12708 induced with CA (resulting in upregulation of Bai enzymes) and then incubated with isoCDCA [143]. While trace levels of isoLCA (<1%) have been reported in vitro during BA metabolism by *C. scindens* ATCC 35704 [144], this may be due to the minor promiscuity known for some bacterial HSDHs [96]. Iso-secondary BAs (e.g., isoDCA and isoLCA) are second only to DCA and LCA in abundance in stool [109] and are less toxic than LCA and DCA to intestinal bacteria [17]. Iso-BA epimerizing HSDHs also show substrate specificity preference toward secondary BAs [18]. It is, therefore, hypothesized that isoLCA and isoDCA are generated from LCA and DCA, respectively, in the gastrointestinal tract. A third point is that enrichment of primary oxo- and β-hydroxy-BAs comes at the expense of primary BAs such as CA and CDCA, which induce expression of the *bai* operon [145]. Indeed, culture-based studies indicate that *C. scindens* VPI 12708 is capable of converting 3,7-dioxocholanoic acid and 7-oxoLCA to LCA only if the cells were preincubated with CA [143].

Numerous gut bacteria, including *Bacteroides* spp. and *E. coli*, encode 7α-HSDH and produce 7-oxo-BAs that are released into the lumen [105,146,147]. The formation of 7-oxo-primary BAs precludes 7α/β-dehydration by the *bai* pathway and must be reduced to proceed. It is, therefore, not surprising that BA 7α-dehydroxylating bacteria express NADP-dependent 7α-HSDH [114]. The BA 7α-HSDH is predicted to be important both in regulating the NAD(H)-dependent BA 7α-dehydroxylating pathway intracellularly and in reducing 7-oxo-BAs imported from the environment.

BA 7α-dehydroxylating bacteria also encode BA 12α-HSDH [23,148]. The formation of 12-oxo-BAs reduces toxicity of BAs toward gut bacteria [7], which is likely why a wide diversity of gut bacteria encode 12α-HSDH [18,23,143,149]. However, substrate specificity of 12α-HSDHs in 7α-dehydroxylating bacteria favors the reductive direction, converting 12-oxoLCA to DCA [23]. We, therefore, hypothesize that BA 7α-dehydroxylating bacteria express BA 12α-HSDH principally to “retoxify” 12-oxoLCA that was generated by bacteria less resistant to DCA.

**Figure 4:**
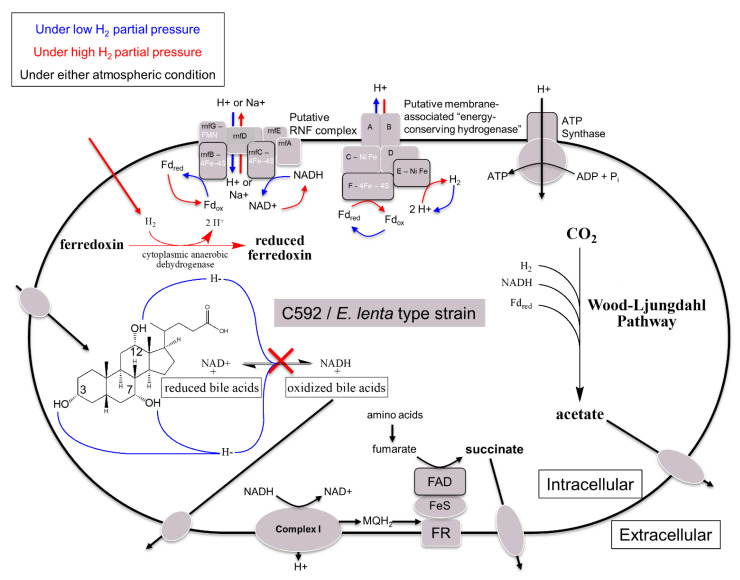
Proposed model for the role of *Eggerthella lenta* hydroxysteroid dehydrogenases: bile acid oxidation provides reductant for the Wood–Ljundahl Pathway (WLP) of acetogenesis. This model is based on biochemical and genomic data demonstrating that *E. lenta* strains contain complete WLP genes, and that bile acid oxidation is inhibited by a hydrogen gas atmosphere.

We recently demonstrated extensive oxidation of BAs by *Eggerthella lenta* [143]. Indeed, *E. lenta* strains C592 and DSM 2243 encode 3α-, 3β-, 7α-, and 12α-HSDHs capable of converting CA to trioxo-cholanoic acid under a nitrogen or carbon dioxide atmosphere. However, BA oxidation was inhibited under a hydrogen gas atmosphere (Figure 4). Genomic analysis revealed genes encoding energy conserving hydrogenase (*echABCDEF*) and Rnf complex (*rnfABCDEG*), as well as a complete Wood–Ljungdahl pathway, suggesting that *E. lenta* is an acetogen [143,150]. The classical acetogen fixes CO_2_ or CO in the presence of H_2_ [151]; however, acetogens are known to utilize a wide range of electron donors. Under this scheme, *E. lenta* HSDH enzymes are hypothesized to generate NADH by oxidizing BAs, which provides reducing equivalents to fix CO_2_. In the presence of H_2_, *E. lenta* hydrogenases reduce NAD^+^ via molecular hydrogen, and BA oxidation is prevented. Additional studies will be needed to confirm this hypothesis linking BA metabolism and H_2_ partial pressure in a novel acetogen.

The role of microbial BA HSDHs in host physiology is also relatively unclear. While the involvement of oxo- and β-BAs in host signaling pathways has not been fully explored, there is evidence that products in the iso-BA pathway activate various host receptors. For example, along with LCA, 3-oxoLCA has been shown to activate the BA receptors FXR, VDR, and PXR [8,56]. In contrast, 12-oxoLCA, 7-oxoLCA, and UDCA did not efficiently activate either FXR or VDR [8,50]. Recently, 3-oxoLCA and a planar iso-BA, isoalloLCA, were shown to be regulators of interleukin (IL)-17a expressing T helper cells (T_H_17) and regulatory T cells (T_reg_) in mice [152]. Determining the full spectrum of both primary and secondary oxo- and β-derivatives against BA-responsive nuclear and G protein-coupled receptors will be important future work.

## 4. Glucocorticoid Metabolism

### 4.1. Host Glucocorticoid Synthesis

Glucocorticoids are involved in diverse essential physiological processes throughout the body [153]. Cortisol and corticosterone are the primary C_21_ glucocorticoids present in humans. However, cortisol concentrations are about 10 times greater than corticosterone [154]. Cortisol plays a major role in the stress response and maintenance of blood glucose concentration, as well as in inhibition of protein synthesis in muscle, of lipogenesis in fat cells, and of the immune system [155].

Cortisol is synthesized in the adrenal gland from cholesterol and involves the action of both cytochrome P450 enzymes and hydroxysteroid dehydrogenases, much like BA biosynthesis (Figure 2). The first step is catalyzed by CYP11A1, which side-chain cleaves cholesterol and results in pregnenolone [9]. This is the rate-limiting step and precursor to many other steroid hormones, including progesterone, corticosterone, aldosterone, testosterone, and estradiol [156]. 17α-Hydroxyprogesterone is then produced by CYP17A1 (17-hydroxylase/17,20 lyase) and HSD3B2 (3β-HSD/Δ^5/4^-isomerase type 2). CYP21A2 converts 17α-hydroxyprogesterone to 11-deoxycortisol. The last reaction results in the formation of cortisol through the action of CYP11B1 [9,10]. Cortisol circulates in serum at concentrations between 100 and 600 nM [9]. Cortisol then acts in peripheral tissues by binding to the nuclear glucocorticoid receptor, resulting in regulation of numerous genes, including those involved in inflammation, immune function, and gluconeogenesis. Cortisol can also bind to mineralocorticoid receptor, which regulates electrolyte balance [157,158]. Cortisol concentrations are tightly regulated by 11β-HSD isoforms 1 and 2. 11β-HSD1/2 interconvert cortisol (C-11 hydroxyl) to its inactive form, cortisone (C-11 ketone), which cannot bind the glucocorticoid receptor or mineralocorticoid receptor. 11β-HSD1 functions primarily as a reductase to activate cortisol in the liver, muscle, and bone. In contrast, 11β-HSD2 acts as a dehydrogenase, inactivating cortisol to cortisone in the kidney, colon, and salivary glands [9].

Human tissues metabolize cortisol in various ways, leading to its excretion primarily in urine. However, low levels of cortisol and its derivatives are secreted in bile and enter the gut [159]. Cortisol undergoes 5α- or 5β-reduction in the liver, while cortisone is only 5β-reduced [160]. After 3α-reduction, 5α/β-tetrahydrocortisol and tetrahydrocortisone are produced, which are the main metabolites of cortisol and cortisone in urine, respectively [9]. Cortisol can also be metabolized by 20α- and 20β-HSDs, yielding either 20α- or 20β-dihydrocortisol [161]. Carbonyl reductase-1 (CBR1) has 20β-HSD activity producing 20β-dihydrocortisol, while a host 20α-HSD has been observed with specificity for progesterone, but not cortisol [9,162]. 20α/β-Reduction of tetrahydrocortisol and tetrahydrocortisone results in α/β-cortols or α/β-cortolones [163].

### 4.2. Host Androgen Synthesis

Androgens are important for metabolic homeostasis and reproductive function in men, as well as women. Androgens are C_19_ steroids that are synthesized in the Leydig cells of the testes or adrenal glands [164]. The primary active androgens in circulation are testosterone and dihydrotestosterone, although, in the adrenal glands, the major products are the androgen precursors dehydroepiandrosterone (and its sulfate ester), androstenedione, and 11β-hydroxyandrostenedione (11β-OHAD) [165].

Androgen biosynthesis in the adrenal cortex begins with side-chain cleavage of cholesterol to pregnenolone by CYP11A1. Then, CYP17A1 hydroxylase and 17,20-lyase activities produce dehydroepiandrosterone (DHEA). HSD3B2 (3β-HSD/Δ^5/4^-isomerase type 2) converts DHEA to androstenedione. Alternatively, AKR1C3 (17β-HSD) can produce androstenediol from DHEA, and HSD3B2 then yields testosterone. Androstenedione can be further converted to 11β-OHAD by adrenal-specific CYP11B1 (11β-hydroxylase) [166].

Even though 11β-OHAD makes up a large proportion of adrenal steroidogenesis, it has historically largely been ignored (except in fishes) due to its low androgenic activity [167]. Storbeck et al. (2013) reported that 11β-OHAD leads to the formation of 11-ketotestosterone (11KT) [168], a potent 11-oxygenated C_19_ androgen involved in castration-resistant prostate cancer [169,170] and polycystic ovary syndrome [170,171]. This is important because, although 11β-OHAD is primarily produced in the adrenal glands by CYP11B1, peripheral side-chain cleavage of cortisol to 11β-OHAD also occurs [172]. Peripheral 11β-OHAD is not formed by CYP17A1 [173]. Thus, the enzyme responsible for cortisol-derived 11β-OHAD may be an unknown host enzyme and/or of microbial origin. Intriguingly, 11β-OHAD has been shown to be produced from side-chain cleavage of cortisol by human gut microbiota [14,174,175,176].

Androgens signal throughout the body by binding to androgen receptor (AR) expressed in various cell types, including B cells, T cells, neutrophils, and macrophages [177], as well as colon cancer cell lines [178]. Nuclear AR is a ligand-dependent transcription factor that, when activated by an androgen, regulates expression of cell growth, differentiation, and even carcinogenesis in some cases [179]. Intestinal cells express both nuclear AR and membrane AR [178,179,180]. Importantly, the gut microbiome has evolved enzymes that catalyze many of the same reactions described for host glucocorticoid and androgen metabolism. This indicates that the host endocrine system has interkingdom components in need of further exploration.

### 4.3. Microbial Cortisol Metabolism

The earliest evidence of microbial biotransformation of cortisol was observed when rectal infusion of cortisol in ulcerative colitis patients led to an increase in urinary excretion of 17-ketosteroids [181]. This increase in urinary steroids was not detected when cortisol treatment coincided with oral neomycin [182], suggesting microbial biotransformation of cortisol. Thereafter, side-chain cleavage of cortisol or steroid-17,20-desmolase activity was observed when human fecal samples produced C_19_ steroids after incubation with cortisol [176].

**Figure 5:**
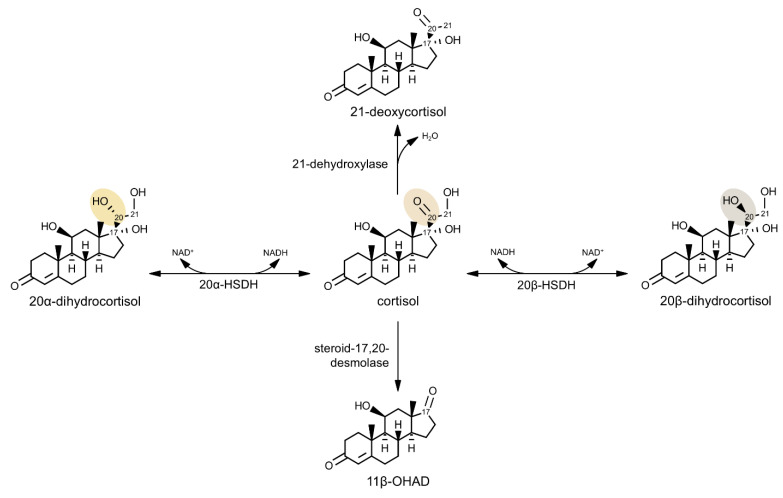
Microbial cortisol hydroxysteroid dehydrogenase metabolism. Cortisol can be reversibly biotransformed by 20β-hydroxysteroid dehydrogenase (20β-HSDH; DesE) to 20β-dihydrocortisol, or by 20α-HSDH (DesC) to 20α-dihydrocortisol. Steroid-17,20-desmolase (DesAB) converts cortisol to 11β-hydroxyandrostenedione (11β-OHAD). 21-Dehydroxylase catalyzes conversion of cortisol to 21-deoxycortisol.

In 1984, a bacterium was isolated from human fecal material exhibiting steroid-17,20-desmolase activity producing 11β-OHAD from cortisol (Figure 2) [174,175]. This organism was named *Clostridium scindens*, formerly *Clostridium* strain 19, which also has BA 7α-dehydroxylation activity [4]. Additional organisms with steroid-17,20-desmolase activity were then isolated: *Butyricicoccus desmolans* ATCC 43058 (formerly *Eubacterium desmolans*), *C. cadaveris* AGR2141 [183], and the urinary microbe *Propionimicrobium lymphophilum* ACS-093-V-SCH5 [184,185]. The operon encoding this activity (*desABCD*) has since been identified by performing RNA-Seq after inducing *C. scindens* ATCC 35704 with cortisol [14]. The inducible *desABCD* operon consists of steroid-17,20-desmolase (DesAB) encoded by *desAB*, a 20α-HSDH (DesC), and a putative transporter (DesD) (Figure 5) [14,186]. *C. scindens* ATCC 35704 DesAB was determined to be a heterotetramer and recognized both cortisol and 11-deoxycortisol, which only differs from cortisol in the absence of an 11β-hydroxyl group [186].

C-20 reduced metabolites of cortisol have been observed in human urine, likely attributable to host enzymes that produce 20α- or 20β-dihydrocortisol and their derivatives [163,187]. However, Winter et al. (1982) showed that gut microbiota can reduce cortisol to 20β-dihydrocortisol, exhibiting 20β-HSDH (DesE) activity [188]. *B. desmolans* and *C cadaveris* express 20β-HSDH [183], along with *Bifidobacterium adolescentis* [188]. Additionally, the gut microbe *Clostridium scindens* ATCC 35704 can convert cortisol to 20α-dihydrocortisol [174]. Thus, gut microbiota encode 20α- and 20β-HSDHs that biotransform cortisol (Figure 5).

Human gut microbiota are also capable of 21-dehydroxylation of corticosteroids. 21-Dehydroxylase activity was first detected in *Eggerthella lenta* (formerly *Eubacterium lentum*) [189,190]. *E. lenta* 21-dehydroxylase has substrate specificity for 11-deoxycorticosterone, deoxycortisol, dehydrocorticosterone, and corticosterone [191,192]. The enzyme requires NAD(P)H and flavin or only reduced flavin mononucleotide for activity [192]. Although this enzyme seems to be specific for corticosterone, 21-dehydroxylation of cortisol to 21-deoxycortisol also occurs [176]. Interestingly, 21-deoxycortisol is a substrate for 11β-HSD2 [193] while the 21-dehydroxylation product of corticosterone is a potent inhibitor [194].

### 4.4. Microbial Cortisol Hydroxysteroid Dehydrogenases

Host hydroxysteroid dehydrogenases have been established as important for biosynthesis and modulation of steroid hormones such as androgens, estrogens, and glucocorticoids for years [5]. Since the discovery of steroid hormone-converting HSDHs in the human gut microbiome, gut bacteria have been proposed to play an important role beyond that of the host in modification of steroids [14]. Within the steroid-17,20-desmolase pathway, two HSDHs have been identified that convert cortisol to 20α- or 20β-dihydrocortisol and may act as enzymatic switches to control formation of 11β-OHAD (Figure 5).

20β-Dihydrocortisol is excreted in urine at rates comparable to that of free cortisol in healthy individuals [161,187]. Urinary excretion of 20α-dihydrocortisol occurs at rates of about 1.5 times the excretion of cortisol [161,187]. Although the physiologic role of 20α- and 20β-dihydrocortisol is not extensively studied, they are elevated in patients with Cushing’s syndrome [187], as well as in patients with hypertension [195].

One of the first organisms studied expressing 20β-HSDH activity was the soil microbe *Streptomyces hydrogenans* [196]. This enzyme reacted with not only cortisol, but also cortisone, cortexolone (lacks C-11 oxygen group), and their 21-aldehydes [196]. More recently, the genes encoding 20β-HSDH in *B. desmolans* and *C. cadaveris*, organisms that were previously shown to have this activity in culture, have been identified [183,184]. The gene is denoted *desE* due to its involvement in the DesAB pathway and because it forms an operon with the *desAB* genes [14,184]. Both *B. desmolans* and *C. cadaveris* are capable of cortisol side-chain cleavage, as well as 20β-oxidoreduction [183,184]. 20β-HSDH has been characterized in detail from *B. desmolans* ATCC 43058, which exhibits specificity for cortisol as substrate and is NAD(H)-dependent [184]. *Bifidobacterium scardovii* ATCC BAA-773 and the urinary tract microbe *Propionimicrobium lymphophilum* ACS-093-V-SCH5 also express 20β-HSDH according to HPLC [184], and *P. lymphophilum* has also been shown to encode *desAB* [184,185]. Additionally, the SDR family NAD(H)-dependent 20β-HSDH product of *desE* in *B. adolescentis* strain L2-32 has been characterized. It is specific for cortisol and was crystallized in both the apo-form without any binding and the binary form with NADH bound at 2.2 and 2.0 Å, respectively [15].

Thus far, 20α-HSDH activity seems to be significantly less widespread than 20β-HSDH, with only one organism shown to exhibit the activity [14,197]. Reduction of cortisol at the C-20 position to 20α-dihydrocortisol was observed in pure cultures of *C. scindens* along with steroid-17,20-desmolase activity [175]. 20α-HSDH from *C. scindens* ATCC 35704 was initially characterized from cell extracts and shown to be NAD(H)-dependent [198]. The gene for 20α-HSDH was identified in 2013 after RNA-Seq analysis revealed a cortisol-inducible operon including *desAB* and *desC*, encoding steroid-17,20-desmolase and 20α-HSDH, respectively [14]. Recently, the *C. scindens* ATCC 35704 20α-HSDH was crystallized for further characterization of the enzymatic mechanism. Hybrid quantum mechanical molecular modeling simulations revealed a reaction mechanism involving a multistep proton relay, which was validated by site-directed mutagenesis experiments of active site and substrate binding residues [16]. An amino-acid homology search based on *C. scindens* ATCC 35704 20α-HSDH within the National Center for Biotechnology Information (NCBI) database uncovered two additional organisms, *Denitratisoma oestradiolicum* DSM 16959 and *Intestinibacillus* sp. Marseille-P4005, which may express 20α-HSDH, although activity has not yet been confirmed [24].

Microbial 20α- and 20β-HSDH may be important regulators of the steroid-17,20-desmolase/DesAB pathway. By competing for cortisol as substrate with DesAB, they would decrease the potential for 11β-OHAD formation. Microbial steroid-17,20-desmolase activity may be one of the important missing enzymes contributing to peripheral 11β-OHAD production in the body [199]. Recent work showed that *Clostridium scindens* ATCC 35704 and the urinary microbe *Propionimicrobium lymphophilum* ACS-093-V-SCH5 can side-chain cleave both cortisol and glucocorticoid drugs [185], suggesting microbial production of 11β-OHAD may occur in both the gut and urinary tract. As mentioned above, 11β-OHAD can be further converted to highly androgenic 11KT [168]. This has compelling implications for androgen-dependent diseases, such as castration-resistant prostate cancer, or diseases defined by androgen excess, such as polycystic ovary syndrome [170]. Further studies are necessary to assess the efficacy of utilizing 20α- and/or 20β-HSDH to mediate 11β-OHAD formation in vivo.

## 5. Conclusions

Overall, both host and microbial HSDHs play pivotal roles in BA and glucocorticoid metabolism. Research on the importance of HSDH-derived BAs on host physiology is in its infancy. However, the immense diversity of these BA metabolites, due to combinations of HSDH activity, means that the gut harbors a multitude of potential candidates for host receptor signaling. Gut microbial cortisol HSDHs are likely important regulators of steroid-17,20-desmolase activity, although additional research is needed to ascertain the physiological significance of 20α- and 20β-HSDH products. New microbial HSDHs are continually being discovered and characterized, which will allow mechanistic study of their impacts in disease models.

Microbial HSDHs may have potential as therapeutic modulators in diseases such as colorectal cancer, liver cancer, castration-resistant prostate cancer, and polycystic ovary syndrome. However, to work toward therapeutics, we must first connect HSDH function to host phenotypes through mechanistic experiments, such as gnotobiotic animal studies [200,201]. Such avenues include developing genetic knockouts of HSDHs in microbes naturally encoding them or, when genetic systems are unavailable, engineering genetically tractable microbes to encode HSDHs. Furthermore, crystal structures of microbial HSDHs will aid in any necessary mutagenesis to rationally design substrate specificity for these enzymes. Integrating functional studies, genetic manipulation, structural biology, and gnotobiotic animal experiments will be imperative to reach a clearer picture of microbial steroid metabolism in the future.

## Data Availability

Not applicable.

## Abbreviations

Bile acid (BA), hydroxysteroid dehydrogenase (HSDH), nicotinamide adenine dinucleotide (phosphate) (NAD(P)(H)), short-chain dehydrogenase/reductase (SDR), medium-chain dehydrogenase/reductase (MDR), aldo-keto reductase (AKR), cytochrome P450 7α-hydroxylase (CYP7A1), 3β-hydroxy-Δ5-C27-steroid oxidoreductase (C27 3β-HSD), 12α-hydroxylase (CYP8B1), 27-hydroxylase (CYP27A1), coenzyme A (CoA), cholic acid (CA), chenodeoxycholic acid (CDCA), farnesoid X receptor (FXR), small heterodimer partner (SHP), liver-related homolog-1 (LRH-1), hepatocyte nuclear factor 4α (HNF4α), fibroblast growth factor 19 (FGF19), fibroblast growth factor receptor 4 (FGFR4), c-jun N-terminal kinase (JNK), pregnane X receptor (PXR), vitamin D receptor (VDR), Takeda G-protein receptor 5 (TGR5), epidermal growth factor receptor (EGFR), bile salt hydrolase (BSH), deoxycholic acid (DCA), lithocholic acid (LCA), BA-inducible (bai), American Type Culture Collection (ATCC), ursodeoxycholic acid (UDCA), 7α,12β-dihydroxy-androsta-1,4-diene-3,17-dione (12β-DHADD), interleukin (IL)-17a-expressing T helper cells (TH17), regulatory T cells (Treg), 17-hydroxylase/17,20 lyase (CYP17A1), 3β-HSD/Δ5/4-isomerase type 2 (HSD3B2), carbonyl reductase 1 (CBR1), 11β-hydroxyandrostenedione (11β-OHAD), dehydroepiandrosterone (DHEA), 11β-hydroxylase (CYP11B1), 11-ketotestosterone (11KT), androgen receptor (AR), steroid-17,20-desmolase (DesAB), 20α-HSDH (DesC), putative cortisol transporter (DesD), 20β-HSDH (DesE), National Center for Biotechnology Information (NCBI).

## References

[1] Norman A.W., Henry H.L., Norman A.W., Henry H.L. (2015). Steroid Hormones: Chemistry, Biosynthesis, and Metabolism. Hormones.

[2] Litwack G., Litwack G. (2018). Steroid hormones. Human Biochemistry.

[3] Payne A.H., Hales D.B. (2004). Overview of steroidogenic enzymes in the pathway from cholesterol to active steroid hormones. Endocr. Rev..

[4] Ridlon J.M., Harris S.C., Bhowmik S., Kang D.J., Hylemon P.B. (2016). Consequences of bile salt biotransformations by intestinal bacteria. Gut Microbes.

[5] Penning T.M. (1997). Molecular endocrinology of hydroxysteroid dehydrogenases. Endocr. Rev..

[6] Hofmann A.F., Roda A. (1984). Physicochemical properties of bile acids and their relationship to biological properties: An overview of the problem. J. Lipid Res..

[7] Watanabe M., Fukiya S., Yokota A. (2017). Comprehensive evaluation of the bactericidal activities of free bile acids in the large intestine of humans and rodents. J. Lipid Res..

[8] Makishima M., Lu T.T., Xie W., Whitfield G.K., Domoto H., Evans R.M., Haussler M.R., Mangelsdorf D.J. (2002). Vitamin D receptor as an intestinal bile acid sensor. Science.

[9] Schiffer L., Barnard L., Baranowski E.S., Gilligan L.C., Taylor A.E., Arlt W., Shackleton C.H.L., Storbeck K.H. (2019). Human steroid biosynthesis, metabolism and excretion are differentially reflected by serum and urine steroid metabolomes: A comprehensive review. J. Steroid Biochem. Mol. Biol..

[10] Miller W.L., Auchus R.J. (2011). The molecular biology, biochemistry, and physiology of human steroidogenesis and its disorders. Endocr. Rev..

[11] Agarwal A.K., Auchus R.J. (2005). Minireview: Cellular redox state regulates hydroxysteroid dehydrogenase activity and intracellular hormone potency. Endocrinology.

[12] Penning T.M., Wangtrakuldee P., Auchus R.J. (2019). Structural and functional biology of aldo-keto reductase steroid-transforming enzymes. Endocr. Rev..

[13] Kenny D.J., Plichta D.R., Shungin D., Koppel N., Hall A.B., Fu B., Vasan R.S., Shaw S.Y., Vlamakis H., Balskus E.P. (2020). Cholesterol metabolism by uncultured human gut bacteria influences host cholesterol level. Cell Host Microbe.

[14] Ridlon J.M., Ikegawa S., Alves J.M.P., Zhou B., Kobayashi A., Iida T., Mitamura K., Tanabe G., Serrano M., De Guzman A. (2013). *Clostridium scindens*: A human gut microbe with a high potential to convert glucocorticoids into androgens. J. Lipid Res..

[15] Doden H.L., Pollet R.M., Mythen S.M., Wawrzak Z., Devendran S., Cann I., Koropatkin N.M., Ridlon J.M. (2019). Structural and biochemical characterization of 20β-hydroxysteroid dehydrogenase from *Bifidobacterium adolescentis* strain L2-32. J. Biol. Chem..

[16] Bernardi R., Doden H., Melo M., Devendran S., Pollet R., Mythen S., Bhowmik S., Lesley S., Cann I., Luthey-Schulten Z. (2020). Bacteria on steroids: The enzymatic mechanism of an NADH-dependent dehydrogenase that regulates the conversion of cortisol to androgen in the gut microbiome. bioRxiv.

[17] Devlin A.S., Fischbach M.A. (2015). A biosynthetic pathway for a prominent class of microbiota-derived bile acids. Nat. Chem. Biol..

[18] Mythen S.M., Devendran S., Méndez-García C., Cann I., Ridlon J.M. (2018). Targeted synthesis and characterization of a gene cluster encoding NAD(P)H-dependent 3α-, 3β-, and 12α-hydroxysteroid dehydrogenases from *Eggerthella* CAG:298, a gut metagenomic sequence. Appl. Env. Microbiol..

[19] Filling C., Berndt K.D., Benach J., Knapp S., Prozorovski T., Nordling E., Ladenstein R., Jörnvall H., Oppermann U. (2002). Critical residues for structure and catalysis in short-chain dehydrogenases/reductases. J. Biol. Chem..

[20] Kallberg Y., Oppermann U., Jörnvall H., Persson B. (2002). Short-chain dehydrogenases/reductases (SDRs). Coenzyme-based functional assignments in completed genomes. Eur. J. Biochem..

[21] Rossmann M.G., Moras D., Olsen K.W. (1974). Chemical and biological evolution of a nucleotide-binding protein. Nature.

[22] Kavanagh K.L., Jörnvall H., Persson B., Oppermann U. (2008). The SDR superfamily: Functional and structural diversity within a family of metabolic and regulatory enzymes. Cell. Mol. Life Sci..

[23] Doden H., Sallam L.A., Devendran S., Ly L., Doden G., Daniel S.L., Alves J.M.P., Ridlon J.M. (2018). Metabolism of oxo-bile acids and characterization of recombinant 12α-hydroxysteroid dehydrogenases from bile acid 7α-dehydroxylating human gut bacteria. Appl. Env. Microbiol..

[24] Doden H., Alves J.M.P., Ridlon J.M. (2020). Identification and characterization of a gene encoding NADP(H)-dependent bile acid 12β-hydroxysteroid dehydrogenase from *Clostridium paraputrificum* ATCC 25780. Biorxiv.

[25] Persson B., Hedlund J., Jornvall H. (2008). The MDR superfamily. Cell. Mol. Life Sci..

[26] Nordling E., Jornvall H., Persson B. (2002). Medium-chain dehydrogenases/reductases (MDR): Family characterizations including genome comparisons and active site modelling. Eur. J. Biochem..

[27] Knoll M., Pleiss J. (2008). The Medium-Chain Dehydrogenase/Reductase Engineering Database: A systematic analysis of a diverse protein family to understand sequence-structure-function relationship. Protein Sci..

[28] Jez J.M., Bennett M.J., Schlegel B.P., Lewis M., Penning T.M. (1997). Comparative anatomy of the aldo-keto reductase superfamily. Biochem. J..

[29] Khanna M., Qin K.N., Wang R.W., Cheng K.C. (1995). Substrate specificity, gene structure, and tissue-specific distribution of multiple human 3α-hydroxysteroid dehydrogenases. J. Biol. Chem..

[30] Zhang Y., Dufort I., Rheault P., Luu-The V. (2000). Characterization of a human 20α-hydroxysteroid dehydrogenase. J. Mol. Endocrinol..

[31] Vlahcevic Z.R., Heuman D.M., Hylemon P.B., Zakim D., Boyer T. (1996). Physiology and pathophysiology of enterohepatic circulation of bile acids. Hepatology: A Textbook of Liver Disease.

[32] Hofmann A.F., Hagey L.R. (2014). Key discoveries in bile acid chemistry and biology and their clinical applications: History of the last eight decades. J. Lipid Res..

[33] Hylemon P.B., Zhou H., Pandak W.M., Ren S., Gil G., Dent P. (2009). Bile acids as regulatory molecules. J. Lipid Res..

[34] Russell D.W. (2003). The enzymes, regulation, and genetics of bile acid synthesis. Annu. Rev. Biochem..

[35] Pandak W.M., Kakiyama G. (2019). The acidic pathway of bile acid synthesis: Not just an alternative pathway. Liver Res..

[36] Schwarz M., Björkhem I., Wright A.C., Davis D.L., Nazer H., Björkhem I., Russell D.W. (2000). The bile acid synthetic gene 3β-hydroxy-Δ5-C27-steroid oxidoreductase is mutated in progressive intrahepatic cholestasis. J. Clin. Investig..

[37] Chiang J.Y.L. (2013). Bile acid metabolism and signaling. Compr. Physiol..

[38] Steinberg S.J., Wang S.J., Kim D.G., Mihalik S.J., Watkins P.A. (1999). Human very-long-chain acyl-CoA synthetase: Cloning, topography, and relevance to branched-chain fatty acid metabolism. Biochem. Biophys. Res. Commun..

[39] Steinberg S.J., Wang S.J., McGuinness M.C., Watkins P.A. (1999). Human liver-specific very-long-chain acyl-Coenzyme A synthetase: cDNA cloning and characterization of a second enzymatically active protein. Mol. Genet. Metab..

[40] Falany C.N., Johnson M.R., Barnes S., Diasio R.B. (1994). Glycine and taurine conjugation of bile acids by a single enzyme: Molecular cloning and expression of human liver bile acid CoA:amino acid N- acyltransferase. J. Biol. Chem..

[41] Hofmann A.F., Hagey L.R., Krasowski M.D. (2010). Bile salts of vertebrates: Structural variation and possible evolutionary significance. J. Lipid Res..

[42] Goto T., Holzinger F., Hagey L.R., Cerrè C., Ton-Nu H.T., Schteingart C.D., Steinbach J.H., Shneider B.L., Hofmann A.F. (2003). Physicochemical and physiological properties of 5α-cyprinol sulfate, the toxic bile salt of cyprinid fish. J. Lipid Res..

[43] Chiang J.Y.L. (2009). Bile acids: Regulation of synthesis. J. Lipid Res..

[44] Craddock A.L., Love M.W., Daniel R.W., Kirby L.C., Walters H.C., Wong M.H., Dawson P.A. (1998). Expression and transport properties of the human ileal and renal sodium- dependent bile acid transporter. Am. J. Physiol..

[45] Dawson P.A., Hubbert M., Haywood J., Craddock A.L., Zerangue N., Christian W.V., Ballatori N. (2005). The heteromeric organic solute transporter α-β, Ostα-Ostβ, is an ileal basolateral bile acid transporter. J. Biol. Chem..

[46] Ballatori N., Christian W.V., Lee J.Y., Dawson P.A., Soroka C.J., Boyer J.L., Madejczyk M.S., Li N. (2005). OSTα-OSTβ: A major basolateral bile acid and steroid transporter in human intestinal, renal, and biliary epithelia. Hepatology.

[47] Kullak-Ublick G.A., Stieger B., Meier P.J. (2004). Enterohepatic bile salt transporters in normal physiology and liver disease. Gastroenterology.

[48] Rembacz K.P., Woudenberg J., Hoekstra M., Jonkers E.Z., Van Den Heuvel F.A.J., Buist-Homan M., Woudenberg-Vrenken T.E., Rohacova J., Marin M.L., Miranda M.A. (2010). Unconjugated bile salts shuttle through hepatocyte peroxisomes for taurine conjugation. Hepatology.

[49] Makishima M., Okamoto A.Y., Repa J.J., Tu H., Learned R.M., Luk A., Hull M.V., Lustig K.D., Mangelsdorf D.J., Shan B. (1999). Identification of a nuclear receptor for bile acids. Science.

[50] Parks D.J., Blanchard S.G., Bledsoe R.K., Chandra G., Consler T.G., Kliewer S.A., Stimmel J.B., Willson T.M., Zavacki A.M., Moore D.D. (1999). Bile acids: Natural ligands for an orphan nuclear receptor. Science.

[51] Goodwin B., Jones S.A., Price R.R., Watson M.A., McKee D.D., Moore L.B., Galardi C., Wilson J.G., Lewis M.C., Roth M.E. (2000). A regulatory cascade of the nuclear receptors FXR, SHP-1, and LRH-1 represses bile acid biosynthesis. Mol. Cell.

[52] Zhang M., Chiang J.Y.L. (2001). Transcriptional regulation of the human sterol 12α-hydroxylase gene (CYP8B1): Roles of hepatocyte nuclear factor 4α in mediating bile acid repression. J. Biol. Chem..

[53] Del Castillo-Olivares A., Campos J.A., Pandak W.M., Gil G. (2004). The role of α1-fetoprotein transcription factor/LRH-1 in bile acid biosynthesis: A known nuclear receptor activator that can act as a suppressor of bile acid biosynthesis. J. Biol. Chem..

[54] Holt J.A., Luo G., Billin A.N., Bisi J., McNeill Y.Y., Kozarsky K.F., Donahee M., Wang D.Y., Mansfield T.A., Kliewer S.A. (2003). Definition of a novel growth factor-dependent signal cascade for the suppression of bile acid biosynthesis. Genes Dev..

[55] Song K.H., Li T., Owsley E., Strom S., Chiang J.Y.L. (2009). Bile acids activate fibroblast growth factor 19 signaling in human hepatocytes to inhibit cholesterol 7α-hydroxylase gene expression. Hepatology.

[56] Staudinger J.L., Goodwin B., Jones S.A., Hawkins-Brown D., MacKenzie K.I., LaTour A., Liu Y., Klaassen C.D., Brown K.K., Reinhard J. (2001). The nuclear receptor PXR is a lithocholic acid sensor that protects against liver toxicity. Proc. Natl. Acad. Sci. USA.

[57] Li T., Chiang J.Y.L. (2005). Mechanism of rifampicin and pregnane X receptor inhibition of human cholesterol 7α-hydroxylase gene transcription. Am. J. Physiol. Gastrointest. Liver Physiol..

[58] Han S., Chiang J.Y.L. (2009). Mechanism of vitamin D receptor inhibition of cholesterol 7α-hydroxylase gene transcription in human hepatocytes. Drug Metab. Dispos..

[59] Keitel V., Cupisti K., Ullmer C., Knoefel W.T., Kubitz R., Häussinger D. (2009). The membrane-bound bile acid receptor TGR5 is localized in the epithelium of human gallbladders. Hepatology.

[60] Ward J.B.J., Mroz M.S., Keely S.J. (2013). The bile acid receptor, TGR5, regulates basal and cholinergic-induced secretory responses in rat colon. Neurogastroenterol. Motil..

[61] Alemi F., Poole D.P., Chiu J., Schoonjans K., Cattaruzza F., Grider J.R., Bunnett N.W., Corvera C.U. (2013). The receptor TGR5 mediates the prokinetic actions of intestinal bile acids and is required for normal defecation in mice. Gastroenterology.

[62] Hegyi P., Maléth J., Walters J.R., Hofmann A.F., Keely S.J. (2018). Guts and gall: Bile acids in regulation of intestinal epithelial function in health and disease. Physiol. Rev..

[63] Yasuda H., Hirata S., Inoue K., Mashima H., Ohnishi H., Yoshiba M. (2007). Involvement of membrane-type bile acid receptor M-BAR/TGR5 in bile acid-induced activation of epidermal growth factor receptor and mitogen-activated protein kinases in gastric carcinoma cells. Biochem. Biophys. Res. Commun..

[64] Qiao L., Studer E., Leach K., McKinstry R., Gupta S., Decker R., Kukreja R., Valerie K., Nagarkatti P., El Deiry W. (2001). Deoxycholic acid (DCA) causes ligand-independent activation of epidermal growth factor receptor (EGFR) and FAS receptor in primary hepatocytes: Inhibition of EGFR/mitogen-activated protein kinase-signaling module enhances DCA-induced apoptosis. Mol. Biol. Cell.

[65] Coleman J.P., Hudson L.L. (1995). Cloning and characterization of a conjugated bile acid hydrolase gene from *Clostridium perfringens*. Appl. Env. Microbiol..

[66] Kishinaka M., Umeda A., Kuroki S. (1994). High concentrations of conjugated bile acids inhibit bacterial growth of *Clostridium perfringens* and induce its extracellular cholylglycine hydrolase. Steroids.

[67] Stellwag E.J., Hylemon P.B. (1976). Purification and characterization of bile salt hydrolase from *Bacteroides fragilis* subsp. fragilis. Biochim. Biophys. Acta.

[68] Yao L., Seaton S.C., Ndousse-Fetter S., Adhikari A.A., Dibenedetto N., Mina A.I., Banks A.S., Bry L., Devlin A.S. (2018). A selective gut bacterial bile salt hydrolase alters host metabolism. Elife.

[69] Elkins C.A., Moser S.A., Savage D.C. (2001). Genes encoding bile salt hydrolases and conjugated bile salt transporters in *Lactobacillus johnsonii* 100-100 and other *Lactobacillus* species. Microbiology.

[70] Tanaka H., Hashiba H., Kok J., Mierau I. (2000). Bile salt hydrolase of *Bifidobacterium longum*-Biochemical and genetic characterization. Appl. Env. Microbiol..

[71] Kim G.B., Miyamoto C.M., Meighen E.A., Lee B.H. (2004). Cloning and characterization of the bile salt hydrolase genes (*bsh*) from *Bifidobacterium bifidum* strains. Appl. Environ. Microbiol..

[72] Wijaya A., Hermann A., Abriouel H., Specht I., Yousif N.M.K., Holzapfel W.H., Franz C.M.A.P. (2004). Cloning of the bile salt hydrolase (*bsh*) gene from *Enterococcus faecium* FAIR-E 345 and chromosomal location of *bsh* genes in food Enterococci. J. Food Prot..

[73] Jones B.V., Begley M., Hill C., Gahan C.G.M., Marchesi J.R. (2008). Functional and comparative metagenomic analysis of bile salt hydrolase activity in the human gut microbiome. Proc. Natl. Acad. Sci. USA.

[74] Ridlon J.M., Kang D.-J., Hylemon P.B. (2006). Bile salt biotransformations by human intestinal bacteria. J. Lipid Res..

[75] Quinn R.A., Melnik A.V., Vrbanac A., Fu T., Patras K.A., Christy M.P., Bodai Z., Belda-Ferre P., Tripathi A., Chung L.K. (2020). Global chemical effects of the microbiome include new bile-acid conjugations. Nature.

[76] Grill J.P., Cayuela C., Antoine J.M., Schneider F. (2000). Isolation and characterization of a *Lactobacillus amylovorus* mutant depleted in conjugated bile salt hydrolase activity: Relation between activity and bile salt resistance. J. Appl. Microbiol..

[77] Mead G.C. (1971). The amino acid-fermenting Clostridia. J. Gen. Microbiol..

[78] Kitahara M., Takamine F., Imamura T., Benno Y. (2000). Assignment of *Eubacterium* sp. VPI 12708 and related strains with high bile acid 7α-dehydroxylating activity to *Clostridium scindens* and proposal of *Clostridium hylemonae* sp. nov., isolated from human faeces. Int. J. Syst. Evol. Microbiol..

[79] Kitahara M., Takamine F., Imamura T., Benno Y. (2001). *Clostridium hiranonis* sp. nov., a human intestinal bacterium with bile acid 7α-dehydroxylating activity. Int. J. Syst. Evol. Microbiol..

[80] Chen X.J., Wang Z.Q., Zhou Z.Y., Zeng N.Y., Huang Q.F., Wang Z.W., Tang W.L., Zhou H.W. (2020). Characterization of *Peptacetobacter hominis* gen. nov., sp. nov., isolated from human faeces, and proposal for the reclassification of *Clostridium hiranonis* within the genus *Peptacetobacter*. Int. J. Syst. Evol. Microbiol..

[81] Franco P., Porru E., Fiori J., Gioiello A., Cerra B., Roda G., Caliceti C., Simoni P., Roda A. (2019). Identification and quantification of oxo-bile acids in human faeces with liquid chromatography–mass spectrometry: A potent tool for human gut acidic sterolbiome studies. J. Chromatogr. A.

[82] Kakiyama G., Muto A., Takei H., Nittono H., Murai T., Kurosawa T., Hofmann A.F., Pandak W.M., Bajaj J.S. (2014). A simple and accurate HPLC method for fecal bile acid profile in healthy and cirrhotic subjects: Validation by GC-MS and LC-MS. J. Lipid Res..

[83] Hylemon P.B., Melone P.D., Franklund C.V., Lund E., Bjorkhemt I. (1991). Mechanism of intestinal 7α-dehydroxylation of cholic acid: Evidence that allo-deoxycholic acid is an inducible side-product. J. Lipid Res..

[84] Ridlon J.M., Kang D.J., Hylemon P.B. (2010). Isolation and characterization of a bile acid inducible 7α-dehydroxylating operon in *Clostridium hylemonae* TN271. Anaerobe.

[85] Mallonee D.H., Hylemon P.B. (1996). Sequencing and expression of a gene encoding a bile acid transporter from *Eubacterium* sp. strain VPI 12708. J. Bacteriol..

[86] Mallonee D.H., Adams J.L., Hylemon P.B. (1992). The bile acid-inducible *baiB* gene from *Eubacterium* sp. strain VPI 12708 encodes a bile acid-coenzyme A ligase. J. Bacteriol..

[87] Mallonee D.H., Lijewski M.A., Hylemon P.B. (1995). Expression in *Escherichia coli* and characterization of a bile acid-inducible 3α-hydroxysteroid dehydrogenase from *Eubacterium* sp. strain VPI 12708. Curr. Microbiol..

[88] Coleman J.P., White W.B., Lijewski M., Hylemon P.B. (1988). Nucleotide sequence and regulation of a gene involved in bile acid 7-dehydroxylation by *Eubacterium* sp. strain VPI 12708. J. Bacteriol..

[89] Gopal-Srivastava R., Mallonee D.H., White W.B., Hylemon P.B. (1990). Multiple copies of a bile acid-inducible gene in *Eubacterium* sp. strain VPI 12708. J. Bacteriol..

[90] White W.B., Franklund C.V., Coleman J.P., Hylemon P.B. (1988). Evidence for a multigene family involved in bile acid 7-dehydroxylation in *Eubacterium* sp. strain VPI 12708. J. Bacteriol..

[91] Devendran S., Shrestha R., Alves J.M.P., Wolf P.G., Ly L., Hernandez A.G., Fields C.J., Daniel S.L., Ridlon M. (2019). *Clostridium scindens* ATCC 35704: Integration of nutritional requirements, the complete genome sequence, and global transcriptional responses to bile acids. Appl. Environ. Microbiol..

[92] Kang D.J., Ridlon J.M., Moore D.R., Barnes S., Hylemon P.B. (2008). *Clostridium scindens baiCD* and *baiH* genes encode stereo-specific 7α/7β-hydroxy-3-oxo-Δ4-cholenoic acid oxidoreductases. Biochim. Biophys. Acta.

[93] Ridlon J.M., Hylemon P.B. (2012). Identification and characterization of two bile acid coenzyme A transferases from *Clostridium scindens*, a bile acid 7α-dehydroxylating intestinal bacterium. J. Lipid Res..

[94] Dawson J.A., Mallonee D.H., Bjorkhem I., Hylemon B.P. (1996). Expression and characterization of a C24 bile acid 7α-dehydratase from *Eubacterium* sp. strain VPI 12708 in *Escherichia coli*. J. Lipid Res..

[95] Ridlon J.M., Bajaj J.S. (2015). The human gut sterolbiome: Bile acid-microbiome endocrine aspects and therapeutics. Acta Pharm. Sin. B.

[96] Harris S.C., Devendran S., Alves J.M.P., Mythen S.M., Hylemon P.B., Ridlon J.M. (2018). Identification of a gene encoding a flavoprotein involved in bile acid metabolism by the human gut bacterium *Clostridium scindens* ATCC 35704. Biochim. Biophys. Acta- Mol. Cell Biol. Lipids.

[97] Funabashi M., Grove T.L., Wang M., Varma Y., McFadden M.E., Brown L.C., Guo C., Higginbottom S., Almo S.C., Fischbach M.A. (2020). A metabolic pathway for bile acid dehydroxylation by the gut microbiome. Nature.

[98] Bhowmik S., Jones D.H., Chiu H.P., Park I.H., Chiu H.J., Axelrod H.L., Farr C.L., Tien H.J., Agarwalla S., Lesley S.A. (2014). Structural and functional characterization of BaiA, an enzyme involved in secondary bile acid synthesis in human gut microbe. Proteins Struct. Funct. Bioinforma..

[99] Heinken A., Ravcheev D.A., Baldini F., Heirendt L., Fleming R.M.T., Thiele I. (2019). Systematic assessment of secondary bile acid metabolism in gut microbes reveals distinct metabolic capabilities in inflammatory bowel disease. Microbiome.

[100] Kurdi P., Kawanishi K., Mizutani K., Yokota A. (2006). Mechanism of growth inhibition by free bile acids in Lactobacilli and Bifidobacteria. J. Bacteriol..

[101] Hofmann A.F., Sjovall J., Kurz G., Radominska A., Schteingart C.D., Tint G.S., Vlahcevic Z.R., Setchell K.D.R. (1992). A proposed nomenclature for bile acids. J. Lipid Res..

[102] Eyssen H., De Pauw G., Stragier J., Verhulst A. (1983). Cooperative formation of ω-muricholic acid by intestinal microorganisms. Appl. Env. Microbiol..

[103] Macdonald I.A., Jellett J.F., Mahony D.E., Holdeman L.V. (1979). Bile salt 3α- and 12α-hydroxysteroid dehydrogenases from *Eubacterium lentum* and related organisms. Appl. Env. Microbiol..

[104] Yoshimoto T., Higashi H., Kanatani A., Lin X.S., Nagai H., Oyama H., Kurazono K., Tsuru D. (1991). Cloning and sequencing of the 7α-hydroxysteroid dehydrogenase gene from *Escherichia coli* HB101 and characterization of the expressed enzyme. J. Bacteriol..

[105] Bennett M.J., McKnight S.L., Coleman J.P. (2003). Cloning and characterization of the NAD-dependent 7α-hydroxysteroid dehydrogenase from Bacteroides fragilis. Curr. Microbiol..

[106] Wang D.Q.H., Carey M.C. (2014). Therapeutic uses of animal biles in traditional Chinese medicine: An ethnopharmacological, biophysical chemical and medicinal review. World J. Gastroenterol..

[107] Hagey L.R., Crombie D.L., Espinosa E., Carey M.C., Igimi H., Hofmann A.F. (1993). Ursodeoxycholic acid in the Ursidae: Biliary bile acids of bears, pandas, and related carnivores. J. Lipid Res..

[108] Goossens J.F., Bailly C. (2019). Ursodeoxycholic acid and cancer: From chemoprevention to chemotherapy. Pharmacol. Ther..

[109] Hamilton J.P., Xie G., Raufman J.P., Hogan S., Griffin T.L., Packard C.A., Chatfield D.A., Hagey L.R., Steinbach J.H., Hofmann A.F. (2007). Human cecal bile acids: Concentration and spectrum. Am. J. Physiol. Gastrointest. Liver Physiol..

[110] Coleman J.P., Hudson L.L., Adams M.J. (1994). Characterization and regulation of the NADP-linked 7α-hydroxysteroid dehydrogenase gene from *Clostridium sordellii*. J. Bacteriol..

[111] Macdonald L.A., Rochon Y.P., Hutchison D.M., Holdeman L.V. (1982). Formation of ursodeoxycholic acid from chenodeoxycholic acid by a 7β-hydroxysteroid dehydrogenase-elaborating *Eubacterium aerofaciens* strain cocultured with 7α-hydroxysteroid dehydrogenase-elaborating organisms. Appl. Env. Microbiol..

[112] Edenharder R., Pfützner A., Hammann R. (1989). Characterization of NAD-dependent 3α- and 3β-hydroxysteroid dehydrogenase and of NADP-dependent 7β-hydroxysteroid dehydrogenase from *Peptostreptococcus productus*. Biochim. Biophys. Acta.

[113] Liu L., Aigner A., Schmid R.D. (2011). Identification, cloning, heterologous expression, and characterization of a NADPH-dependent 7β-hydroxysteroid dehydrogenase from *Collinsella aerofaciens*. Appl. Microbiol. Biotechnol..

[114] Baron S.F., Franklund C.V., Hylemon P.B. (1991). Cloning, sequencing, and expression of the gene coding for bile acid 7α-hydroxysteroid dehydrogenase from *Eubacterium* sp. strain VPI 12708. J. Bacteriol..

[115] Sutherland J.D., Williams C.N. (1985). Bile acid induction of 7α-and 7β-hydroxysteroid dehydrogenases in *Clostridium limosum*. J. Lipid Res..

[116] Macdonald I.A., Meier E.C., Mahony D.E., Costain G.A. (1976). 3α-, 7α- And 12α-hydroxysteroid dehydrogenase activities from *Clostridium perfringens*. Biochim. Biophys. Acta.

[117] Bernstein C., Holubec H., Bhattacharyya A.K., Nguyen H., Payne C.M., Zaitlin B., Bernstein H. (2011). Carcinogenicity of deoxycholate, a secondary bile acid. Arch. Toxicol..

[118] Cao H., Xu M., Dong W., Deng B., Wang S., Zhang Y., Wang S., Luo S., Wang W., Qi Y. (2017). Secondary bile acid-induced dysbiosis promotes intestinal carcinogenesis. Int. J. Cancer.

[119] Yoshimoto S., Loo T.M., Atarashi K., Kanda H., Sato S., Oyadomari S., Iwakura Y., Oshima K., Morita H., Hattori M. (2013). Obesity-induced gut microbial metabolite promotes liver cancer through senescence secretome. Nature.

[120] Ma C., Han M., Heinrich B., Fu Q., Zhang Q., Sandhu M., Agdashian D., Terabe M., Berzofsky J.A., Fako V. (2018). Gut microbiome–mediated bile acid metabolism regulates liver cancer via NKT cells. Science.

[121] Magouliotis D.E., Tasiopoulou V.S., Svokos A.A., Svokos K.A., Chatedaki C., Sioka E., Zacharoulis D. (2017). Ursodeoxycholic acid in the prevention of gallstone formation after bariatric surgery: An updated systematic review and meta-analysis. Obes. Surg..

[122] Kim E.K., Cho J.H., Kim E.J., Kim Y.J. (2017). Ursodeoxycholic acid inhibits the proliferation of colon cancer cells by regulating oxidative stress and cancer stem-like cell growth. PLoS ONE.

[123] Lazaridis K.N., Gores G.J., Lindor K.D. (2001). Ursodeoxycholic acid “mechanisms of action and clinical use in hepatobiliary disorders”. J. Hepatol..

[124] White B.A., Fricke R.J., Hylemon P.B. (1982). 7β-Dehydroxylation of ursodeoxycholic acid by whole cells and cell extracts of the intestinal anaerobic bacterium, *Eubacterium* species V.P.I 12708. J. Lipid Res..

[125] Macdonald I.A., Hutchison D.M. (1982). Epimerization versus dehydroxylation of the 7α-hydroxyl- group of primary bile acids: Competitive studies with *Clostridium absonum* and 7α-dehydroxylating bacteria (*Eubacterium* sp.). J. Steroid Biochem..

[126] Kotb M.A. (2012). Molecular mechanisms of ursodeoxycholic acid toxicity & side effects: Ursodeoxycholic acid freezes regeneration & induces hibernation mode. Int. J. Mol. Sci..

[127] Edenharder R., Pfützner M., Hammann R. (1989). NADP-dependent 3β-, 7α-and 7β-hydroxysteroid dehydrogenase activities from a lecithinase-lipase-negative *Clostridium* species 25.11.c. Biochim. Biophys. Acta.

[128] Marschall H.U., Oppermann U.C.T., Svensson S., Nordling E., Persson B., Höög J.O., Jörnvall H. (2000). Human liver class I alcohol dehydrogenase isozyme: The sole cytosolic 3β-hydroxysteroid dehydrogenase of iso bile acids. Hepatology.

[129] Macdonald I.A., Jellett J.F., Mahony D.E. (1979). 12alpha-Hydroxysteroid dehydrogenase from *Clostridium* group P strain C48-50 ATCC #29733: Partial purification and characterization. J. Lipid Res..

[130] Harris J.N., Hylemon P.B. (1978). Partial purification and characterization of NADP-dependent 12α-hydroxysteroid dehydrogenase from *Clostridium leptum*. Biochim. Biophys. Acta.

[131] Edenharder R., Schneider J. (1985). 12β-Dehydrogenation of bile acids by *Clostridium paraputrificum*, *C. tertium*, and *C. difficile* and epimerization at carbon-12 of deoxycholic acid by cocultivation with 12α-dehydrogenating *Eubacterium lentum*. Appl. Env. Microbiol..

[132] Edenharder R., Pfützner A. (1988). Characterization of NADP-dependent 12β-hydroxysteroid dehydrogenase from *Clostridium paraputrificum*. Biochim. Biophys. Acta.

[133] Fischer S., Ben F., Paumgartner G. (1991). Unchanged levels of keto bile acids in bile after cholecystectomy. Digestion.

[134] Eneroth P., Hellstrom K., Sjovall J. (1968). A method for quantitative determination of bile acid in human feces. Acta Chem. Scand..

[135] Reddy B.S., Maeura Y. (1984). Tumor promotion by dietary fat in azoxymethane-induced colon carcinogenesis in female F344 rats: Influence of amount and source of dietary fat. J. Natl. Cancer Inst..

[136] Eneroth P., Gordon B., Ryhage R., Sjövall J. (1966). Identification of mono- and dihydroxy bile acids in human feces by gas-liquid chromatography and mass spectrometry. J. Lipid Res..

[137] Horinouchi M., Hayashi T., Koshino H., Malon M., Yamamoto T., Kudo T. (2008). Identification of genes involved in inversion of stereochemistry of a C-12 hydroxyl group in the catabolism of cholic acid by *Comamonas testosteroni* TA441. J. Bacteriol..

[138] Holert J., Kulić Ž., Yücel O., Suvekbala V., Suter M.J.F., Möller H.M., Philipp B. (2013). Degradation of the acyl side chain of the steroid compound cholate in *Pseudomonas* sp. strain Chol1 proceeds via an aldehyde intermediate. J. Bacteriol..

[139] Song C., Wang B., Tan J., Zhu L., Lou D. (2017). Discovery of tauroursodeoxycholic acid biotransformation enzymes from the gut microbiome of black bears using metagenomics. Sci. Rep..

[140] Haselwood G.A.D., Wootton V.M. (1951). Comparative studies of “bile salts”. Pythocholic acid. Biochem. J..

[141] Pellicciari R., Gioiello A., Sabbatini P., Venturoni F., Nuti R., Colliva C., Rizzo G., Adorini L., Pruzanski M., Roda A. (2012). Avicholic acid: A lead compound from birds on the route to potent TGR5 modulators. ACS Med. Chem. Lett..

[142] Meigs R.A., Ryan K.J. (1966). 16α-hydroxysteroid dehydrogenase of rat kidney. J. Biol. Chem..

[143] Harris S.C., Devendran S., Méndez- García C., Mythen S.M., Wright C.L., Fields C.J., Hernandez A.G., Cann I., Hylemon P.B., Ridlon J.M. (2018). Bile acid oxidation by *Eggerthella lenta* strains C592 and DSM 2243. Gut Microbes.

[144] Marion S., Studer N., Desharnais L., Menin L., Escrig S., Meibom A., Hapfelmeier S., Bernier-Latmani R. (2019). In vitro and in vivo characterization of *Clostridium scindens* bile acid transformations. Gut Microbes.

[145] White B.A., Lipsky R.L., Fricke R.J., Hylemon P.B. (1980). Bile acid induction specificity of 7α-dehydroxylase activity in an intestinal *Eubacterium* species. Steroids.

[146] Fukiya S., Arata M., Kawashima H., Yoshida D., Kaneko M., Minamida K., Watanabe J., Ogura Y., Uchida K., Itoh K. (2009). Conversion of cholic acid and chenodeoxycholic acid into their 7-oxo derivatives by *Bacteroides intestinalis* AM-1 isolated from human feces. FEMS Microbiol. Lett..

[147] Tanaka N., Nonaka T., Tanabe T., Yoshimoto T., Tsuru D., Mitsui Y. (1996). Crystal structures of the binary and ternary complexes of 7α-hydroxysteroid dehydrogenase from *Escherichia coli*. Biochemistry.

[148] Masuda N., Oda H. (1983). 7α-Dehydroxylation of bile acids by resting cells of an unidentified, Gram-positive, nonsporeforming anaerobic bacterium. Appl. Env. Microbiol..

[149] Kisiela M., Skarka A., Ebert B., Maser E. (2012). Hydroxysteroid dehydrogenases (HSDs) in bacteria—A bioinformatic perspective. J. Steroid Biochem. Mol. Biol..

[150] Hylemon P.B., Harris S.C., Ridlon J.M. (2018). Metabolism of hydrogen gases and bile acids in the gut microbiome. FEBS Lett..

[151] Ragsdale S.W., Pierce E. (2008). Acetogenesis and the Wood–Ljungdahl pathway of CO_2_ fixation. Biochim. Biophys. Acta.

[152] Hang S., Paik D., Yao L., Kim E., Jamma T., Lu J., Ha S., Nelson B.N., Kelly S.P., Wu L. (2019). Bile acid metabolites control T_H_17 and T_reg_ cell differentiation. Nature.

[153] Cain D.W., Cidlowski J.A. (2017). Immune regulation by glucocorticoids. Nat. Rev. Immunol..

[154] Morris D.J. (2015). Why do humans have two glucocorticoids: A question of intestinal fortitude. Steroids.

[155] Norman A.W., Henry H.L., Norman A.W., Henry H.L. (2015). Adrenal Corticoids. Hormones.

[156] Miller W.L. (2017). Steroidogenesis: Unanswered questions. Trends Endocrinol. Metab..

[157] Wang J.-C., Harris C., Wang J.-C., Harris C. (2015). Glucocorticoid Signaling: From Molecules to Mice to Man.

[158] Arriza J.L., Weinberger C., Cerelli G., Glaser T.M., Handelin B.L., Housman D.E., Evans R.M. (1987). Cloning of human mineralocorticoid receptor complementary DNA: Structural and functional kinship with the glucocorticoid receptor. Science.

[159] Peterson R.E., Wyngaarden J.B., Guerra S.L., Brodie B.B., Bunim J.J. (1955). The physiological disposition and metabolic fate of hydrocortisone in man. J. Clin. Investig. Investig..

[160] Gold B.N.I., Smith L.L., Moore F.D. (1959). Cortisol metabolism in man: Observations of pathways, pool sizes of metabolites and rates of formation of metabolites. J. Clin. Investig..

[161] Eisenschmid B., Heilmann P., Oelkers W., Rejaibi R., Schöneshöfer M. (1987). 20-Dihydroisomers of cortisol and cortisone in human urine: Excretion rates under different physiological conditions. J. Clin. Chem. Clin. Biochem..

[162] Morgan R.A., Beck K.R., Nixon M., Homer N.Z.M., Crawford A.A., Melchers D., Houtman R., Meijer O.C., Stomby A., Anderson A.J. (2017). Carbonyl reductase 1 catalyzes 20β-reduction of glucocorticoids, modulating receptor activation and metabolic complications of obesity. Sci. Rep..

[163] Shackleton C.H.L., Roitman E., Monder C., Bradlow H.L. (1980). Gas chromatographic and mass spectrometric analysis of urinary acidic metabolites of cortisol. Steroids.

[164] Schiffer L., Arlt W., Storbeck K.H. (2018). Intracrine androgen biosynthesis, metabolism and action revisited. Mol. Cell. Endocrinol..

[165] Rege J., Nakamura Y., Satoh F., Morimoto R., Kennedy M.R., Layman L.C., Honma S., Sasano H., Rainey W.E. (2013). Liquid chromatography-tandem mass spectrometry analysis of human adrenal vein 19-carbon steroids before and after ACTH stimulation. J. Clin. Endocrinol. Metab..

[166] Turcu A., Smith J.M., Auchus R., Rainey W.E. (2014). Adrenal androgens and androgen precursors-Definition, synthesis, regulation and physiologic actions. Compr. Physiol..

[167] Pretorius E., Arlt W., Storbeck K.-H. (2017). A new dawn for androgens: Novel lessons from 11-oxygenated C19 steroids. Mol. Cell Endocrinol..

[168] Storbeck K., Bloem L.M., Africander D., Schloms L., Swart P., Swart A.C. (2013). 11β-Hydroxydihydrotestosterone and 11-ketodihydrotestosterone, novel C19 steroids with androgenic activity: A putative role in castration resistant prostate cancer?. Mol. Cell. Endocrinol..

[169] Pretorius E., Africander D.J., Vlok M., Perkins M.S., Quanson J., Storbeck K.-H. (2016). 11-Ketodihydrotestosterone in castration resistant prostate cancer: Potent androgens which can no longer be ignored. PLoS ONE.

[170] Turcu A.F., Auchus R.J. (2017). Clinical significance of 11-oxygenated androgens. Curr. Opin. Endocrinol. Diabetes Obes..

[171] O’Reilly M.W., Kempegowda P., Jenkinson C., Taylor A.E., Quanson J.L., Storbeck K.H., Arlt W. (2017). 11-oxygenated C19 steroids are the predominant androgens in polycystic ovary syndrome. J. Clin. Endocrinol. Metab..

[172] Swart A.C., Storbeck K.H. (2015). 11β-hydroxyandrostenedione: Downstream metabolism by 11βHSD, 17βHSD and SRD5A produces novel substrates in familiar pathways. Mol. Cell. Endocrinol..

[173] Shackleton C.H.L., Neres M.S., Hughes B.A., Stewart P.M., Kater C.E. (2008). 17-Hydroxylase/C17,20-lyase (CYP17) is not the enzyme responsible for side-chain cleavage of cortisol and its metabolites. Steroids.

[174] Winter J., Morris G.N., O’Rourke-Locascio S., Bokkenheuser V.D., Mosbach E.H., Cohen B.I., Hylemon P.B. (1984). Mode of action of steroid desmolase and reductases synthesized by *Clostridium “scindens”* (formerly *Clostridium* strain 19). J. Lipid Res..

[175] Bokkenheuser V.D., Morris G.N., Ritchie A.E., Holdeman L.V., Winter J. (1984). Biosynthesis of androgen from cortisol by a species of *Clostridium* recovered from human fecal flora. J. Infec. Dis..

[176] Cerone-McLernon A.M., Winter J., Mosbach E.H., Bokkenheuser V.D. (1981). Side-chain cleavage of cortisol by fecal flora. Biochim. Biophys. Acta.

[177] Lai J.J., Lai K.P., Zeng W., Chuang K.H., Altuwaijri S., Chang C. (2012). Androgen receptor influences on body defense system via modulation of innate and adaptive immune systems. Am. J. Pathol..

[178] Gu S., Papadopoulou N., Nasir O., Föller M., Alevizopoulos K., Lang F., Stournaras C. (2011). Activation of membrane androgen receptors in colon cancer inhibits the prosurvival signals Akt/Bad in vitro and in vivo and blocks migration via vinculin/actin signaling. Mol. Med..

[179] D’Errico I., Moschetta A. (2008). Nuclear receptors, intestinal architecture and colon cancer: An intriguing link. Cell. Mol. Life Sci..

[180] Catalano M.G., Pfeffer U., Raineri M., Ferro P., Curto A., Capuzzi P., Corno F., Berta L., Fortunati N. (2000). Altered expression of androgen-receptor isoforms in human colon-cancer tissues. Int. J. Cancer.

[181] Nabarro J.D.N., Moxham A., Walker G., Slater J.D.H. (1957). Rectal Hydrocortisone. Br. Med. J..

[182] Wade A.P., Slater J.D., Kellie A.E., Holliday M.E. (1959). Urinary excretion of 17-ketosteroids following rectal infusion of cortisol. J. Clin. Endocrinol. Metab..

[183] Bokkenheuser V.D., Winter J., Morris G.N., Locascio S. (1986). Steroid desmolase synthesis by *Eubacterium desmolans* and *Clostridium cadavaris*. Appl. Env. Microbiol..

[184] Devendran S., Méndez-García C., Ridlon J.M. (2017). Identification and characterization of a 20β-HSDH from the anaerobic gut bacterium *Butyricicoccus desmolans* ATCC 43058. J. Lipid Res..

[185] Ly L.K., Rowles J.L., Paul H.M., Alves J.M.P., Yemm C., Wolf P.M., Devendran S., Hudson M.E., Morris D.J., Erdman J.W. (2020). Bacterial steroid-17,20-desmolase is a taxonomically rare enzymatic pathway that converts prednisone to 1,4-androstanediene-3,11,17-trione, a metabolite that causes proliferation of prostate cancer cells. J. Steroid Biochem. Mol. Biol..

[186] Devendran S., Mythen S.M., Ridlon J.M. (2018). The desA and desB genes from *Clostridium scindens* ATCC 35704 encode steroid-17,20-desmolase. J. Lipid Res..

[187] Schoneshofer M., Weber B., Nigam S. (1983). Increased urinary excretion of free 20α-and 20β-dihydrocortisol in a hypercortisolemic but hypocortisoluric patient with Cushing’s disease. Clin. Chem..

[188] Winter J., Cerone-McLernon A., O’Rourke S., Ponticorvo L., Bokkenheuser V.D. (1982). Formation of 20β-dihydrosteroids by anaerobic bacteria. J. Steroid Biochem..

[189] Bokkenheuser V.D., Winter J., Dehazya P., Kelly W.G. (1977). Isolation and characterization of human fecal bacteria capable of 21-dehydroxylating corticoids. Appl. Environ. Microbiol..

[190] Winter J., Bokkenheuser V.D. (1978). 21-Dehydroxylation of corticoids by anaerobic bacteria isolated from human fecal flora. J. Steroid Biochem..

[191] Feighner S.D., Hylemon P.B. (1980). Characterization of a corticosteroid 21-dehydroxylase from the intestinal anaerobic bacterium, Eubacterium lentum. J. Lipid Res..

[192] Feighner S.D., Bokkenheuser V.D., Winter J., Hylemon P.B. (1979). Characterization of a C21 neutral steroid hormone transforming enzyme, 21-dehydroxylase, in crude cell extracts of *Eubacterium lentum*. Biochim. Biophys. Acta.

[193] Barnard L., Gent R., van Rooyen D., Swart A.C. (2017). Adrenal C11-oxy C21 steroids contribute to the C11-oxy C19 steroid pool via the backdoor pathway in the biosynthesis and metabolism of 21-deoxycortisol and 21-deoxycortisone. J. Steroid Biochem. Mol. Biol..

[194] Latif S.A., Pardo H.A., Hardy M.P., Morris D.J. (2005). Endogenous selective inhibitors of 11β-hydroxysteroid dehydrogenase isoforms 1 and 2 of adrenal origin. Mol. Cell Endocrinol..

[195] Kornel L., Miyabo S., Saito Z., Cha R.-W., Wu F.-T. (1975). Corticosteroids in human blood. VIII. Cortisol metabolites in plasma of normotensive subjects and patients with essential hypertension. J. Clin. Endocrinol. Metab..

[196] Szymanski E.S., Furfine C.S. (1977). 20β-Hydroxysteroid oxidoreductase: Kinetics and binding of corticosteroids and corticosteroid-21-aldehydes. J. Biol. Chem..

[197] Javdan B., Lopez J.G., Chankhamjon P., Lee Y.C.J., Hull R., Wu Q., Wang X., Chatterjee S., Donia M.S. (2020). Personalized mapping of drug metabolism by the human gut microbiome. Cell.

[198] Krafft A.E., Hylemon P.B. (1989). Purification and characterization of a novel form of 20α-hydroxysteroid dehydrogenase from *Clostridium scindens*. J. Bacteriol..

[199] Ly L.K., Doden H.L., Ridlon J.M. (2021). Gut feelings about bacterial steroid-17,20-desmolase. Mol. Cell. Endocrinol..

[200] Koppel N., Balskus E.P. (2016). Exploring and understanding the biochemical diversity of the human microbiota. Cell Chem. Biol..

[201] Fischbach M.A. (2018). Microbiome: Focus on causation and mechanism. Cell.

